# Inflammatory, synaptic, motor, and behavioral alterations induced by gestational sepsis on the offspring at different stages of life

**DOI:** 10.1186/s12974-021-02106-1

**Published:** 2021-02-25

**Authors:** Marcelo Gomes Granja, Letícia Pires Alves, Marina Leardini-Tristão, Michelle Edelman Saul, Letícia Coelho Bortoni, Flávia Maciel de Moraes, Erica Camila Ferreira, Bianca Portugal Tavares de Moraes, Victória Zerboni da Silva, Adrielle Ferreira Ribeiro dos Santos, Adriana Ribeiro Silva, Cassiano Felippe Gonçalves-de-Albuquerque, Victorio Bambini-Junior, Andrew S. Weyrich, Matthew T. Rondina, Guy A. Zimmerman, Hugo Caire de Castro-Faria-Neto

**Affiliations:** 1 Laboratório de Imunofarmacologia, Instituto Oswaldo Cruz, Fundação Oswaldo Cruz – Fiocruz, Rio de Janeiro, Brazil; 2 Programa de Pós-graduação em Biologia Molecular e Celular, Universidade Federal do Estado do Rio de Janeiro – UNIRIO, Rio de Janeiro, Brazil; 3 Faculdade de Medicina, Universidade Estácio de Sá – UNESA, Rio de Janeiro, Brazil; 4 Programa de Pós-graduação em Neurociências, Universidade Federal Fluminense – UFF, Niterói, Rio de Janeiro Brazil; 5 School of Pharmacy and Biomedical Sciences, University of Central Lancashire, PR1 2HE, Lancashire, Preston, England, UK; 6 Department of Internal Medicine and Molecular Medicine Program, University of Utah, Salt Lake City, UT USA; 7 Department of Internal Medicine and Pathology, University of Utah, Salt Lake City, UT USA; 8 Department of Internal Medicine and GRECC, George E. Wahlen VAMC, Salt Lake City, UT USA

**Keywords:** Gestational sepsis, Synaptophysin, Depression, Memory, Motor damage

## Abstract

**Background:**

The term sepsis is used to designate a systemic condition of infection and inflammation associated with hemodynamic changes that result in organic dysfunction. Gestational sepsis can impair the development of the central nervous system and may promote permanent behavior alterations in the offspring. The aim of our work was to evaluate the effects of maternal sepsis on inflammatory cytokine levels and synaptic proteins in the hippocampus, neocortex, frontal cortex, and cerebellum of neonatal, young, and adult mice. Additionally, we analyzed the motor development, behavioral features, and cognitive impairments in neonatal, young and adult offspring.

**Methods:**

Pregnant mice at the 14^th^ embryonic day (E14) were intratracheally instilled with saline 0.9% solution (control group) or *Klebsiella* spp. (3 × 10^8^ CFU) (sepsis group) and started on meropenem after 5 h. The offspring was sacrificed at postnatal day (P) 2, P8, P30, and P60 and samples of liver, lung, and brain were collected for TNF-α, IL-1β, and IL-6 measurements by ELISA. Synaptophysin, PSD95, and β-tubulin levels were analyzed by Western blot. Motor tests were performed at all analyzed ages and behavioral assessments were performed in offspring at P30 and P60.

**Results:**

Gestational sepsis induces a systemic pro-inflammatory response in neonates at P2 and P8 characterized by an increase in cytokine levels. Maternal sepsis induced systemic downregulation of pro-inflammatory cytokines, while in the hippocampus, neocortex, frontal cortex, and cerebellum an inflammatory response was detected. These changes in the brain immunity were accompanied by a reduction of synaptophysin and PSD95 levels in the hippocampus, neocortex, frontal cortex, and cerebellum, in all ages. Behavioral tests demonstrated motor impairment in neonates, and depressive-like behavior, fear-conditioned memory, and learning impairments in animals at P30 and P60, while spatial memory abilities were affected only at P60, indicating that gestational sepsis not only induces an inflammatory response in neonatal mouse brains, but also affects neurodevelopment, and leads to a plethora of behavioral alterations and cognitive impairments in the offspring.

**Conclusion:**

These data suggest that maternal sepsis may be causatively related to the development of depression, learning, and memory impairments in the litter.

**Supplementary Information:**

The online version contains supplementary material available at 10.1186/s12974-021-02106-1.

## Background

Sepsis is a life-threatening clinical syndrome characterized by an exacerbated host inflammatory response to infection [1] and is one of the most common reasons for admission to the intensive care unit (ICU), with approximately 19.4 million new hospitalizations each year, of whom 14.1 million survive to hospital discharge [2]. The mortality rates range from 30 to 60% for sepsis and septic shock [3]. Furthermore, it is estimated that 17% of the patients experience severe and persistent physical, cognitive, and psychological disability and about 30% die during the following year post discharge [2]. Considering the importance of effective identification and management of sepsis, in 2017 the World Health Organization (WHO) included this syndrome in the list of global health priorities [4].

Vertical transmission of sepsis is a concern in pregnant women due to premature rupture of membranes, and contact with bacteria in the maternal urinary tract or in the mother’s endometrium during childbirth [5]. Maternal sepsis is associated with an increased rate of preterm birth, fetal infection, hypoxia, and acidosis and is a risk factor for cesarean section [6]. Studies also indicate that prenatal exposure to infections may affect fetal neurodevelopment and has been associated with higher risks of mental illness such as schizophrenia and autism spectrum disorder (ASD) [7].

The host immune system responds to the invading pathogen with an inflammatory process. When a dysregulated response sets in, a massive increase of pro-inflammatory mediators occurs. This inflammatory process is thought to be directly linked to the sepsis pathophysiology. Some of the most studied pro-inflammatory cytokines in the pathophysiology of sepsis are interleukin-1β (IL-1β) and tumor necrosis factor α (TNF-α), both released from activated macrophages. These cytokines act synergistically and induce changes in vascular permeability, pulmonary edema and hemorrhage [8].

Circulatory dysfunction, inadequate tissue perfusion with a decrease in O_2_ (hypoxia), and in energy supplies are the mechanisms associated with multiple organ dysfunction in sepsis [9], and the brain is the first organ among them to show early signs of dysfunction [10]. Sepsis-associated encephalopathy (SAE) is defined as multifactorial brain dysfunction induced by an inflammatory response to infection [10]. Cognitive impairment may occur in the early or late stages of sepsis as part of SAE. Early stages of SAE usually present as attention lapses, inappropriate behavior, disorientation, and irritability [11]. Delirium, which may be the first manifestation of sepsis, severe agitation, excessive somnolence, stupor, and coma are more often seen in the presence of refractory septic shock. There is convincing evidence that neurological damage may persist even after the recovery from sepsis. Consequently, patients may experience difficulties in social and professional reintegration, compromising the quality of life post sepsis [10].

Because of the difficulty of a specific treatment for SAE, an early detection of the underlying infection, a prompt treatment to eradicate microorganisms, supportive therapies for organs’ dysfunctions, and the withdrawal of drugs with neurotoxic potential are crucial for the management of this complication [12]. Although impairments of motor coordination, cognition, and memory are well-known as long-term consequences in survivors of sepsis in the adult population, this scenario is not clarified in the children at different stages of development. It is still unclear if they are susceptible to the same consequences, if the infants would be able to recover faster, and if they are more easily affected than the adult population. These are a few of the questions that need to be answered to help overcome severe healthcare challenges. In the present study, we investigated effects of maternal sepsis on the inflammatory responses, synaptic regulation, motor coordination, and cognition of the offspring.

## Methods

### Animals

We used pregnant Swiss Webster (SW) mice from the Oswaldo Cruz Foundation breeding unit, Rio de Janeiro, Brazil. Animals were lodged at 22 °C with a 12 h light/dark cycle and with free access to food and water. This study was approved by FIOCRUZ Animal Welfare Committee (CEUA-L018/17). For the neonatal motor tests, we used 10–12 offspring from each litter. We consider one litter as one experimental number. For the behavioral tests, we used 2–3 offspring from each litter. The tests were repeated 3–4 times, using litters from different experiments of sepsis induction with a total of 9–11 animals per group. Western blot (WB) and enzyme-linked immunosorbent assay (ELISA) were performed using samples from 1 to 2 offspring from each litter and repeated 3 times.

### Induction of maternal sepsis

Female SW mice at gestational day 14 (i.e., embryonic day 14) were anesthetized with Isoflurane calibrated at 3.0 MAC dosage (minimum alveolar concentration) by the vaporizer equipment. With the aid of a tracheoscope, we performed an orotracheal intubation followed by injection of 50 μl of sterile saline solution to the animals of the control group, and a suspension containing 3X10^8^ CFU of *Klebsiella pneumoniae* (ATCC 700603 from INCQS-IOC-FIOCRUZ/RJ, Brazil) to the animals of the sepsis group. At the end of the procedure, all animals were maintained with food and water ad libitum. The animals received treatment subcutaneously (s.c.) after 5 h, 24 h, 48 h, and 72 h of the surgical procedure. Meropenem (10 mg/kg) was diluted in saline and administered s.c. in 500 μl per animal. In order to quantify the severity of sepsis, mice were evaluated between 5 and 96 h after the infection based on the following parameters: piloerection, contracted abdomen, changes in locomotion, respiratory rate, lacrimation, grabbing force, turgor, body temperature, interest in the environment, loss of motor activity, and changes in feces. Each animal was scored by our criteria adapted from Brealey et al., Reis et al., and Silva et al. [13–15], and received a total score between 1 and 11. In this evaluation, the higher scores reflect the more severity arbitrarily quantified as 1–3 (mild sepsis), 4–7 (moderate sepsis), and 8–11 (severe sepsis). Only offspring of pregnant mice that received a score of moderate sepsis were used in the experiments.

### Cytokine determination

Placenta, liver, lung, and brain tissues were harvested for cytokine (IL-1β, IL-6, and TNF-α) analysis by ELISA using the duo set kit (R&D Systems, Minneapolis, MN, USA). The tissues were homogenized in a protease inhibiting cocktail containing PBS, 0.1% triton, and protease inhibitor (50 mM—Roche AG, Basel, Switzerland) and then centrifuged (4750 rpm at 4 °C for 20 min). The supernatant was collected and protein content quantified (10× dilution – 10 μl of the sample/90 μl PBS) by Pierce BCA protein assay kit (Thermo Scientific, USA) (standard 2 mg/ml albumin curve - wavelength 562 nm). ELISA (R&D Systems,Minneapolis, MN; IL-1β - MLB00C/IL-6 - M6000B / TNF-α - MTA00B) readings were made at 450 nm on a plate reader.

### Synaptophysin and PSD95 detection

We used Western blot to detect synaptophysin and postsynaptic density protein-95 (PSD95). The hippocampus (i.e., CA1, CA2, and CA3 areas), neocortex (i.e., motor, sensory, and association regions), prefrontal cortex (i.e., all cortex regions from the prefrontal lobe to the central sulcus), and cerebellum (i.e., cerebrocerebellum, spinocerebellum, and the vestibulocerebellum regions) were dissected, macerated, and homogenized at 4 °C in RIPA buffer and phosphatase inhibitor cocktails (Roche). Tissues were stored at − 20 °C for further protein quantification, as described above. Western blot analyses were performed with whole tissues lysates (10 μg of proteins) using anti-synaptophysin (ab32271, 1:40000 dilution, Abcam), anti-PSD-95 (ab18258, 1:1000 dilution, Abcam), and anti-β-tubulin (66240-1-Ig, 1:20.000, Proteintech). Horse anti-mouse IgG antibody (H+L), peroxidase (PI-2000, 1:10.000, Vector Laboratories) and goat anti-rabbit IgG antibody (H+L), peroxidase (PI-1000, 1:10,000 Vector Laboratories) were added to bind to the primary antibody. We performed detection with a dark chamber, using a hypercassette™ (Amersham Pharmacia Biotech, UK). The nitrocellulose membrane was exposed to the luminescent agent ECL (6883S (A: B = 1: 1), Cell Signaling Technology) in a chemiluminescence film (Amersham Hyperfilm™, GE Healthcare limited). Bands were digitized and analyzed by size and intensity with Image J.

### Analyses of motor performance and motor coordination

After the determination of sex as described by Wolterink-Donselaar et al. (2009) [16], we tested male mouse neonates at postnatal day (P) 2 and P8 for the performance of cliff aversion, negative geotaxis, and hindlimb suspension, and older mice at P30 and P60 for the Rota Rod test. The tests were performed in the evening at the same time of the day in order to eliminate circadian differences in behavior. Male pups were carefully removed from the dam for no more than 15 min at the time of measurement to prevent a rapid loss of body temperature and their hunger due to the separation from a mother. All the neonatal motor and neurobehavioral tests were performed as described by Feather-Schussler and Ferguson [17].

#### Cliff aversion

The cliff aversion test was performed at P2 and P8 to evaluate physical strength and coordination [17]. Animals were positioned on the edge of the box, with only the elbows on it, leaving the forelimbs and their noses off the platform. The analysis was performed by measuring the total length for the animal to move the head away from the edge of the box. As the pup’s eyes were still closed at these ages, their fear was not considered as the driving force for the aversion. When the animal fell off the edge, an additional test was performed. When no response was observed after 30 s, the test was interrupted to avoid the learning of the experiment, which would interfere in the analysis, and the animal was considered as failing the test.

#### Negative geotaxis

Negative geotaxis test evaluates the reflex of negative geotaxis and the motor coordination in neonatal mice [17]. Animals were evaluated at P2 and P8. Mice were placed on a − 45° inclined plane with their heads posed downward and were held for 5 s. After the intensity of motion and vestibular reflexes are preserved, the pups were monitored to turn around the face against the slope in a maximum of 2 min time. This taxis response against the gravitation is an innate behavior. We evaluated the length of time required for each animal to complete a 180° turn. Animals that did not fall off the slope or left the upper edge of the tube were eliminated from the test after 3 trials.

#### Hindlimb suspension

Hindlimb suspension test determines the strength of the hindlimbs as well as the neuromuscular function [17]. We designed the test specifically for neonates (2 and 8 days old pups). The animals were suspended by the hocks at the edge of the standard conical 50 ml tube, for evaluating the time for the animal to get off the edge and fall onto the gauze at the bottom of the tube. Animals that did not fall or move off of the edge were eliminated from the test.

#### Rota Rod

We performed the Rota Rod test [18] to evaluate motor ability and motor coordination of male animals at P30 (young) and P60 (adult). The Rota Rod apparatus (OmniRotor, Omnitech Electronics, Inc., Columbus, OH, USA) consists of a cylinder 7 cm away from the surface of the table, rotating at a speed of 4 rpm and acceleration of 20 rpm/min. The locomotive capacity was quantified by measuring the time for the animal to stay on the rotating cylinder [19]. The animals were trained for 2 consecutive days before the experiment. In the training sessions, the animals were kept on the cylinder for 3 min each day, thus providing standard habituation and learning. On the test day, the animals had 3 opportunities to stay on the cylinder for up to 3 min. The time that each animal stayed on the cylinder was recorded during the trials [18].

### Behavioral tests of mature animals

All the behavioral tests were performed in male offspring at P30 and P60.

#### Forced swim test

The test was conducted as previously reported [20]. Male mice were individually forced to swim inside a beaker (height 18 cm; diameter 10 cm) filled in with fresh water at 26 °C with a depth of 12 cm. Animals were placed in the water for 7 min (2 min for habituation, and 5 min for the test session). The total duration of the immobility (passive floating as opposed to struggling, climbing, and diving) for each mouse was manually measured.

#### Morris water maze

Morris water maze was designed as a method to assess spatial learning and memory [21]. The apparatus is a circular black tank (150 cm in diameter and 30 cm high). The tank was filled with water at 26 °C to match the room temperature and the water level was 30 cm in height. A hidden platform (10 cm in diameter) was used as the escape target. Room lighting was indirect. The test consists of two parts: training trials and tests. During four consecutive days of the training trials, the capacity of the animals to learn the spatial location of the hidden platform was evaluated. On the fifth day, the hidden platform was removed, and then, the time spent on the hidden platform quadrant was measured to assess spatial memory as the test paradigm.

#### Fear conditioning memory test

Mice were placed into a specific chamber individually. After 3 min of habituation, mice were subjected to an electric foot-shock (0.75 mA, 3 s) once simultaneously to a tone (2.5 kHz, 85 dB, 3 s) and were then returned to their home cages. On the next day, mice were placed in the same chamber for 3 min and the tone was applied without a foot-shock. The behavior of the mice was recorded by a digital video camera and was manually analyzed. Continuous immobility for > 1 s was defined as freezing behavior, and the total freezing time (i.e., length of time of immobility) was measured [22].

#### Tail suspension test

The tail suspension test is based on observing the escape attempts from an adverse situation. Mice bound with a tape were suspended on a horizontal bar by their tail for 6 min. It is expected for non-affected mice to show agitation (strong shaking of body and moving limbs) because they are trying to escape from the stressful situation. If they give up from the task and become immobile, it could be due to a depressive-like behavior [23]. The test was recorded using a digital video camera, during which the time length of immobility was measured, and then, each mouse was scored by a trained technician.

#### Sucrose preference test

The mice were housed individually with two bottles containing regular water for 24 h. After 24 h, a bottle containing regular water and 1% sucrose solution replaced one bottle of regular water. The positions of two bottles were exchanged 12 h and 24 h later, and then those two bottles were weighed [24]. Sucrose preference was calculated using the following formula [sucrose consumed/(water consumed + sucrose consumed)] for each mouse. Animals were excluded from the experiment if they were found to have liquid in their cages, indicating that the bottles were probably mispositioned.

#### Elevated plus maze test

The elevated plus maze test equipment consists of two opposed open arms and two opposed closed arms, all facing a central platform which was 40 cm elevated from the floor. The apparatus was placed in a small closed room illuminated by a 15-W light (dim environment). Each mouse was placed on the central platform facing one of the closed arms. Mice were allowed to explore the apparatus for 3 min. The number of times each mouse entered into open arms and the time spent in each arm was manually recorded [15, 25, 26].

#### Light/dark box test

The light/dark box test has been shown to reliably predict the anxiolytic and anxiogenic-like effects in rodents. This test has the advantages of a quick and easy performance without prior training of the animals and not requiring food or water deprivation. Transitions in this test are considered an index of activity and exploration because habituation over time is seen with this measure, whereas the time spent in each compartment of light and dark reflects aversion and attraction, respectively.

The light/dark box apparatus consisted of a wooden box (48-cm length × 24-cm height × 27-cm width) divided into two equal size compartments by a barrier that contained a doorway (10-cm height × 10-cm width). One of the compartments was painted black, and it was covered with a lid. The other compartment was painted white and illuminated with a 60-W light bulb positioned 40 cm above the upper edge of the box. The test was performed as described previously by Bourin [27]. On the day of the experiment, we transported the mice to the darkened test room and left in their home cages for 2 h. Then the animals were placed in the middle of the lit compartment, facing away from the dark chamber and were allowed to freely explore the apparatus for 5 min. We recorded the time spent in the lit compartment, the number of entrances into the lit compartment, and latency time to enter (with all four paws) the dark compartment [27, 28].

### Statistical analyses

Clinical scores curves were derived by the Kaplan-Meier method and compared by the log-rank test. Mann-Whitney *U* test was performed for independent samples confirmed by unpaired *t* test. Parameter changes between different groups over time were evaluated by a two-way ANOVA with repeated measurements. The significance level was set at 5%. Data are expressed as means ± SEM, and the differences between groups were considered significant if *p* < 0.05. All tests were performed in GraphPad Prism 6.0 (GraphPad Software, San Diego, CA). The statistical analyses from all figures are reported in Additional File 1.

## Results

### Gestational sepsis is associated with higher miscarriage rate and low-weight offspring

**Fig. 1:**
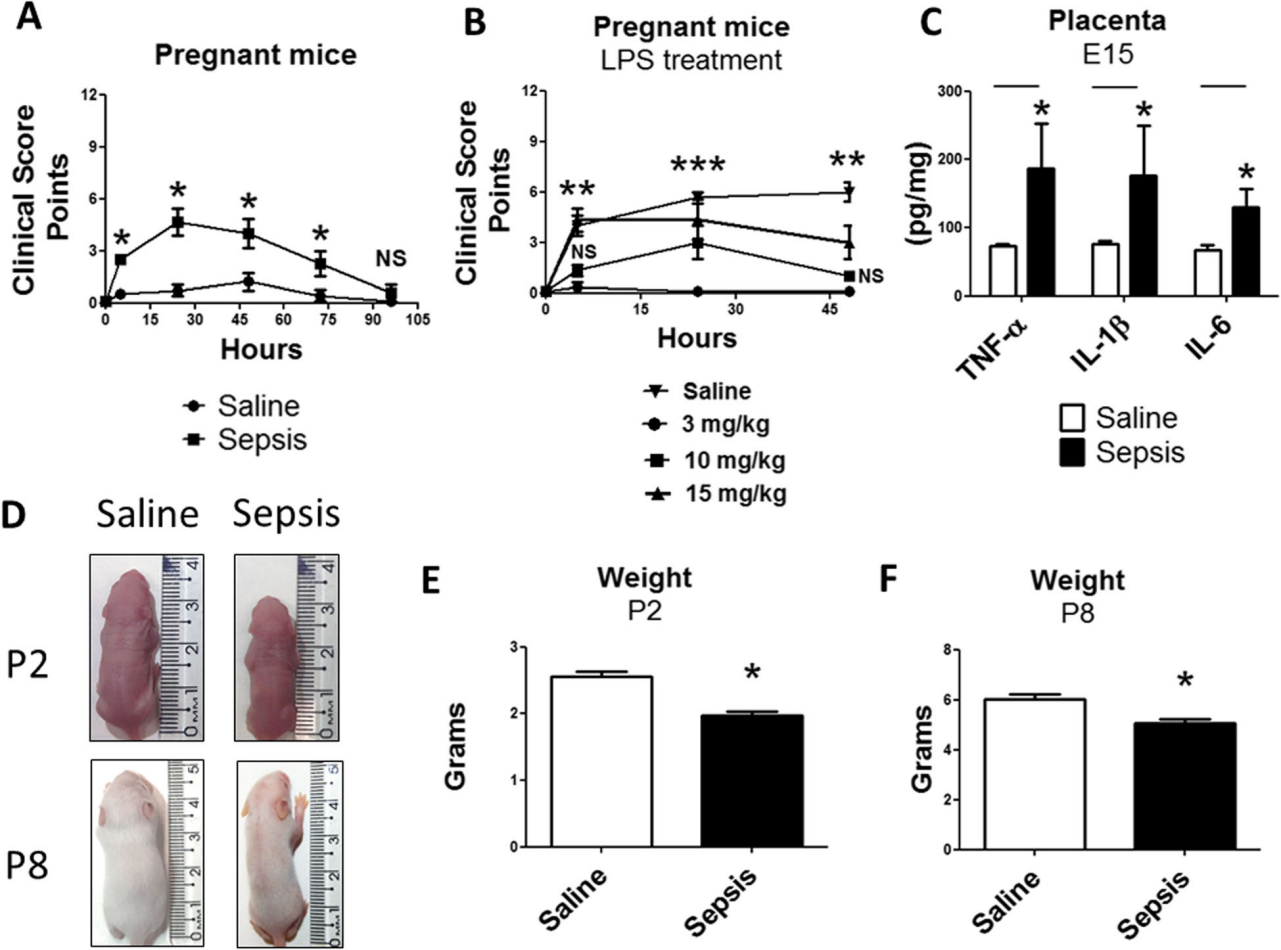
Gestational sepsis causes low-weight offspring. **a** Clinical score evaluation of pregnant mice over time after induction of sepsis at E14 with *Klebsiella pneumoniae* instillation in the sepsis group and instillation of saline solution in the control group. Each point is the mean +/− SEM from at least 12 pregnant mice. **b** LPS solution (3 mg/kg, 10 mg/kg, and 15 mg/kg) and saline solution were administered in pregnant mice at E14. Mice were monitored over 48 h for clinical scores. **c** TNF-α, IL-1β, and IL-6 levels measured by ELISA in the placenta 24 h after induction of sepsis (i.e., E15). Each bar is the mean +/− SEM from 12 placentas. **d** Representative pictures and their body-size of offspring measured at P2 and P8. **e**, **f** Body-weight of offspring measured at P2 (**e**) and P8 (**f**). Each bar is the mean +/− SEM from 20 animals. *p < 0.05 compared saline to sepsis. **p < 0.05 compared 10 mg/kg, and 15 mg/kg to saline. ***p < 0.05 compared 5 mg/kg, 10 mg/kg, and 15 mg/kg to saline. *NS* not significant

The first aim of this study was to evaluate the severity of clinical scores after sepsis induction and antibiotic therapy in pregnant mice. We induced sepsis at embryonic day 14 (E14), and antibiotic treatment was injected subcutaneously in the pregnants after 5 h, 24 h, 48 h, and 72 h of the procedure. We observed an increase in clinical scores as early as 5 h after bacterial challenge, and the highest clinical score was achieved 24 h after the sepsis induction (Fig. 1a). We also compared the clinical scores resulting from *Klebsiella pneumoniae* instillation with that of lipopolysaccharide (LPS 10 mg/kg) injection used to induce an endotoxemia model (Fig. 1b). Our results indicated that the clinical score computed at 24 h after sepsis induction is similar to the score observed after LPS injection (10 mg/kg). Clinical scores then decreased until reaching control values at 4 days of treatment (Fig. 1a). Placental cytokine levels (TNF-α, IL-1β, IL-6) were also increased at 24 h after sepsis induction (Fig. 1c). We also observed that the miscarriage rate was higher in animals exposed to gestational sepsis (Additional file 2). In addition, this model enabled us to rescue pregnant mice from sepsis and to study the offspring for long-term consequences of sepsis. In fact, the pathophysiological effect of offspring was clear immediately after birth, as neonates from septic mothers showed low weight at P2 and P8 (Fig. 1d–f) in comparison to neonates from the control group. However, when we evaluated body weight at P30 and P60, animals from the sepsis group did not show significant differences compared to the control group (Additional file 2).

### Gestational sepsis triggers a neonatal systemic inflammatory response

To determine if gestational sepsis induces upregulation of cytokine up in neonates, we analyzed the levels of TNF-α, IL-1β, and IL-6 in the liver, lung, and brain of neonates at P2 and P8. Mice at P2 presented a systemic pro-inflammatory profile with significant increases in TNF-α, IL-1β, and IL-6 levels in the liver and lung (Additional File 3A and 3B). Later, at P8, all cytokines were returned to basal levels, except TNF-α, which was still increased in the liver (Additional File 3D and 3E). At P2 in the brain, there were also significant increases in TNF-α and IL-1β levels, but not in IL-6. Interestingly, at P8, TNF-α and IL-6 were increased in the brain, but not IL-1β (Additional File 3C and 3F).

### Neonates from septic mothers display motor impairment

**Fig. 2:**
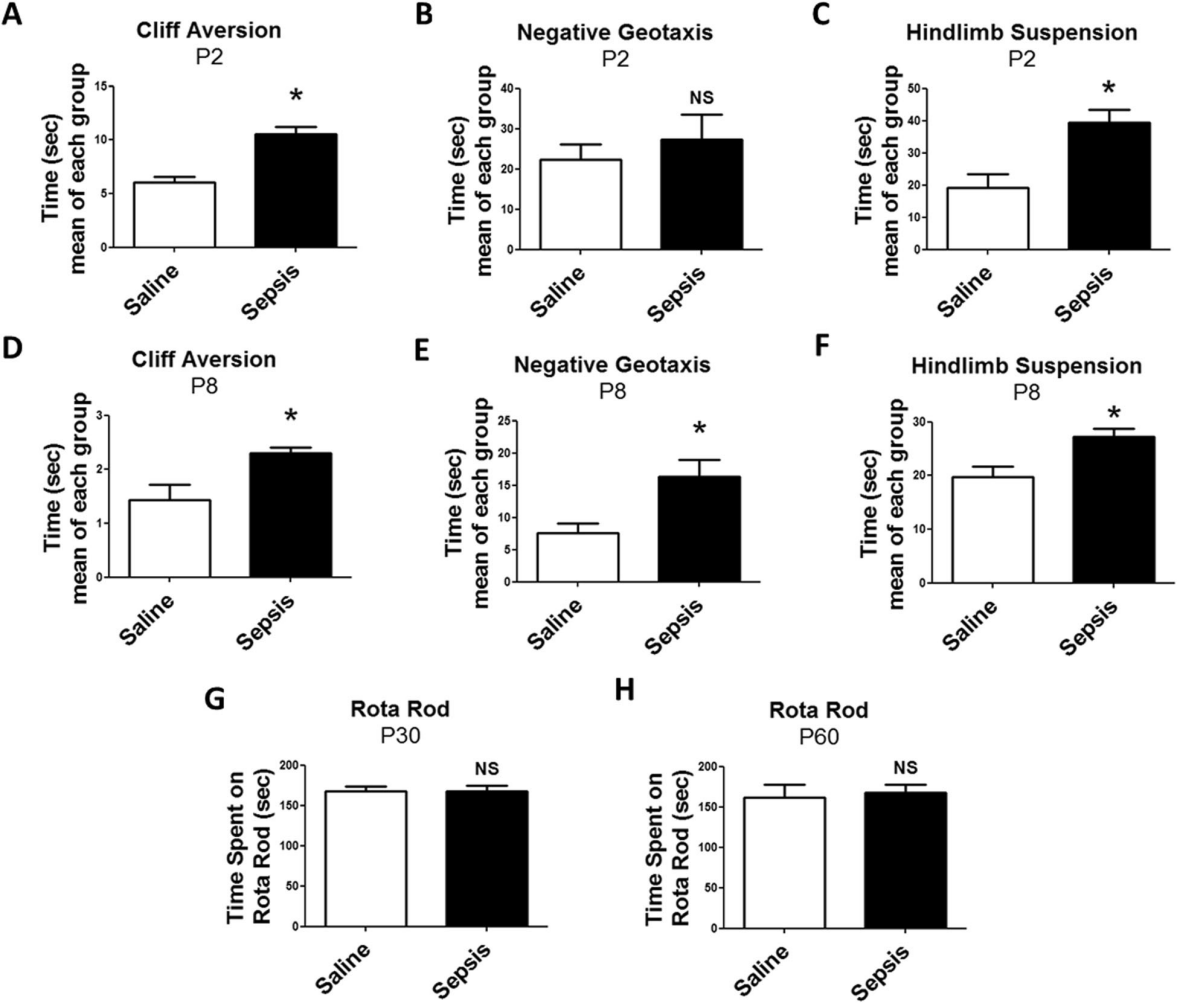
Motor impairments in neonates submitted to gestational sepsis. **a**, **d** For the cliff aversion test, offspring at P2 (**a**) and P8 (**d**) were positioned on the edge of the box and the total time that it took mice to turn away from the cliff and move away from the edge was recorded. Each bar is the mean +/− SEM from 3 litters with 10–12 offspring each. **b, e** The negative geotaxis assesses the motor coordination of animals at P2 (**b**) and P8 (**e**) by comparing the time to complete the test. Each bar is the mean +/− SEM from 3 litters with 10–12 offspring each. **c, f** The hindlimb suspension assesses the animal strength by recording their latency to fall at P2 (**c**) and P8 (**f**). Each bar is the mean +/− SEM from 3 litters with 10–12 offspring each. **g**, **h** The animals were subjected to the Rota Rod test to evaluate the motor ability in young (**g**) and adult (**h**) offspring from mothers submitted to gestational sepsis. Each bar is the mean +/− SEM from 3 litters with 10–12 offspring each. *p < 0.05 compared saline to sepsis. *NS* not significant

To determine if gestational sepsis is associated with motor impairments in the offspring, we performed cliff aversion, negative geotaxis, and hindlimb suspension tests. At P2, offspring born from mothers with gestational sepsis required more time to perform the cliff aversion (Fig. 2a) and hindlimb suspension (Fig. 2c) tests, while there were no significant differences in the negative geotaxis results between the offspring born from septic and control mothers (Fig. 2b).

We next performed the same tests at later phase of neonatal development (i.e., P8). In both the cliff aversion (Fig. 2d) and hindlimb suspension (Fig. 2f) tests, P8 offspring born from mothers with gestational sepsis continued to show reduced abilities in comparison to the control group. At this age, the results from the negative geotaxis test also showed a significant impairment of the performance, which was not seen earlier at P2 (Fig. 2e). These results suggest that gestational sepsis induces the impairment of motor coordination or the reduction of physical strength of neonates. Next, we investigated if the motor impairments detected during neonatal stages were still present in the offspring at P30 (young mice) and P60 (adult mice). Young and adult offspring from the control and sepsis groups showed similar performance in the Rota Rod test (Fig. 2g, h), suggesting that throughout their development, animals regained their motor abilities and coordination.

In the cliff aversion experiments with animals at P2, only two animals from the control group and three animals from the sepsis group failed the test. At P8, only one animal in the control group failed the test. For the negative geotaxis test, three animals at P2 from each group were excluded from the test, and at P8 two animals from the control group and three animals from the sepsis group were removed from the analyses. Evaluating the hindlimb suspension at P2, five animals in the control group and seven animals in the sepsis group were eliminated, while at P8 three saline animals and four sepsis animals were eliminated (data not shown).

### Gestational sepsis induces synaptic and inflammatory alterations in the neocortex, hippocampus, and cerebellum of neonates

**Fig. 3:**
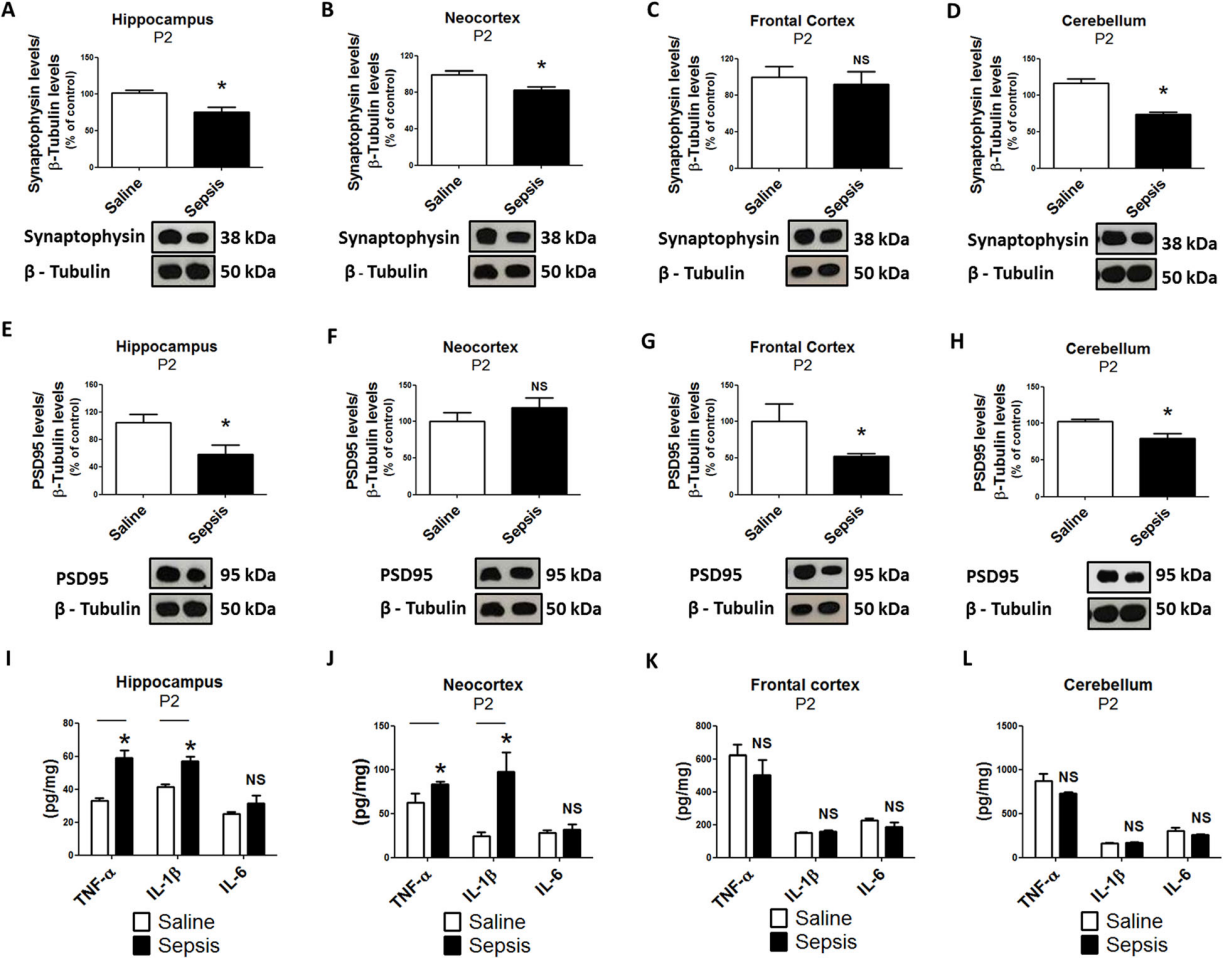
Gestational sepsis downregulates synaptophysin and PSD95 expression, and induces immune activation in neonates’ brains at P2. **a**–**d** Western Blot was performed to detect synaptophysin and β-tubulin in the hippocampus (**a**), neocortex (**b**), frontal cortex (**c**), and cerebellum (**d**). Each bar is the mean +/− SEM from 3 to 7 animals. **e**–**h** Western blot was performed to detect PSD95 and β-tubulin in the hippocampus (**e**), neocortex (**f**), frontal cortex (**g**), and cerebellum (**h**). Each bar is the mean +/− SEM from 3 to 4 animals. **i**–**l** The immune response at P2 was evaluated with TNF-α, IL-1β, and IL-6 levels analyzed by ELISA in the hippocampus (**i**), neocortex **j**), frontal cortex (**k**), and cerebellum (**l**). Each bar is the mean +/− SEM from 3 animals from different litter each one. *p < 0.05 compared saline to sepsis. *NS* not significant

**Fig. 4:**
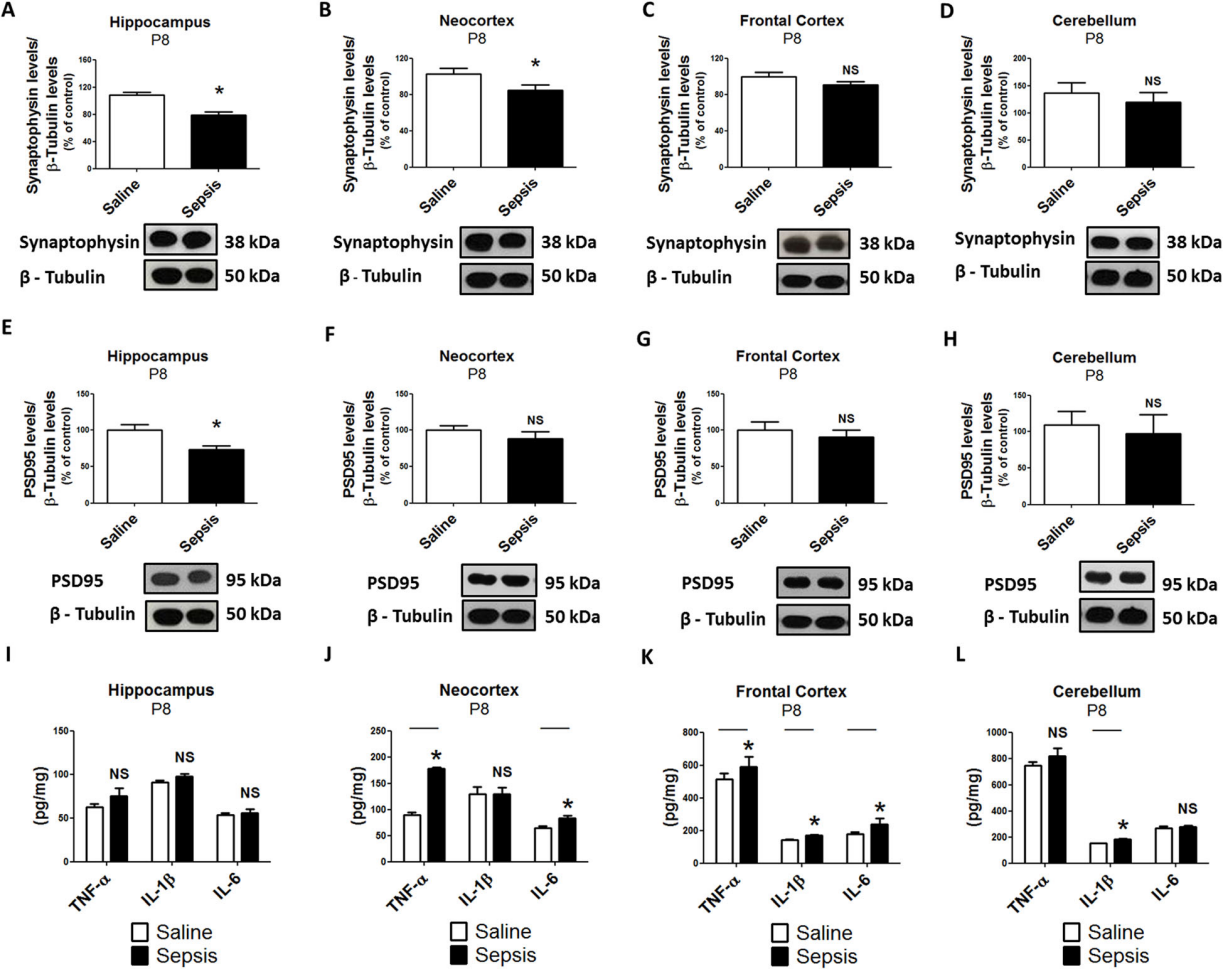
Gestational sepsis downregulates synaptophysin and PSD95 expression, and induces immune activation in neonates’ brains at P8. **a**–**d** Western Blot was performed to detect synaptophysin and β-tubulin in the hippocampus (**a**), neocortex (**b**), frontal cortex (**c**), and cerebellum (**d**). Each bar is the mean +/− SEM from 4 to 6 animals. **e**–**h** Western blot was performed to detect PSD95 and β-tubulin in the hippocampus (**e**), neocortex (**f**), frontal cortex (**g**), and cerebellum (**h**). Each bar is the mean +/− SEM from 3 to 4 animals. (I – L) The immune response at P8 was evaluated through TNF-α, IL-1β, and IL-6 levels analyzed by ELISA in the hippocampus (**i**), neocortex (**j**), frontal cortex (**k**), and cerebellum (**l**). Each bar is the mean +/− SEM from 3 animals from different litter each one. *p < 0.05 compared saline to sepsis. *NS* not significant

Since we observed evidence of an inflammatory response in the brain, along with impaired motor coordination of neonates, we decided to investigate the link between cytokine profiles and the expression of synaptic proteins. In this study, we investigated the presynaptic protein synaptophysin and PSD95 specifically in the hippocampus, neocortex, frontal cortex and cerebellum (Fig. 3). Synaptophysin indispensable membrane molecules for the release of synaptic vesicles and can be used as a presynaptic density marker [29, 30]. PSD95 is a major scaffold protein that regulates central nervous system (CNS) excitatory synapses of postsynaptic density and synaptic maturation by stabilizing glutamate receptors to postsynaptic membranes [31, 32]. As shown in Fig. 3, we found that both synaptic proteins were downregulated in the hippocampus and cerebellum and that TNF-α and IL-1β levels were elevated in the hippocampus at P2 of neonates born following gestational sepsis (Fig. 3a, d, e, h, i, l). In addition, the cytokines were elevated and synaptophysin was also downregulated in the neocortex at the same period. (Fig. 3b, f, j). In the frontal cortex, only PSD95 levels were downregulated (Fig. 3c, g, k). Synaptophysin and PSD95 expression were still below control values in samples of hippocampus at P8 of neonates born from mothers with gestational sepsis (Fig. 4a, e). Synaptophysin expression in the neocortex was downregulated while TNF-α and IL-6 levels were elevated at P8 of animals from the septic group (Fig. 4b, j). The synaptic proteins were not altered in the frontal cortex and cerebellum at P8 (Fig. 4c, d, g, h). Pro-inflammatory cytokines were elevated in these brain regions in neonates at P8 (Fig. 4k, l) with the exception of IL-1β, which was only elevated in the cerebellum in neonates at this age.

### Gestational sepsis impacts on synaptophysin and PSD95 expression and cytokine levels in the adult offspring

**Fig. 5:**
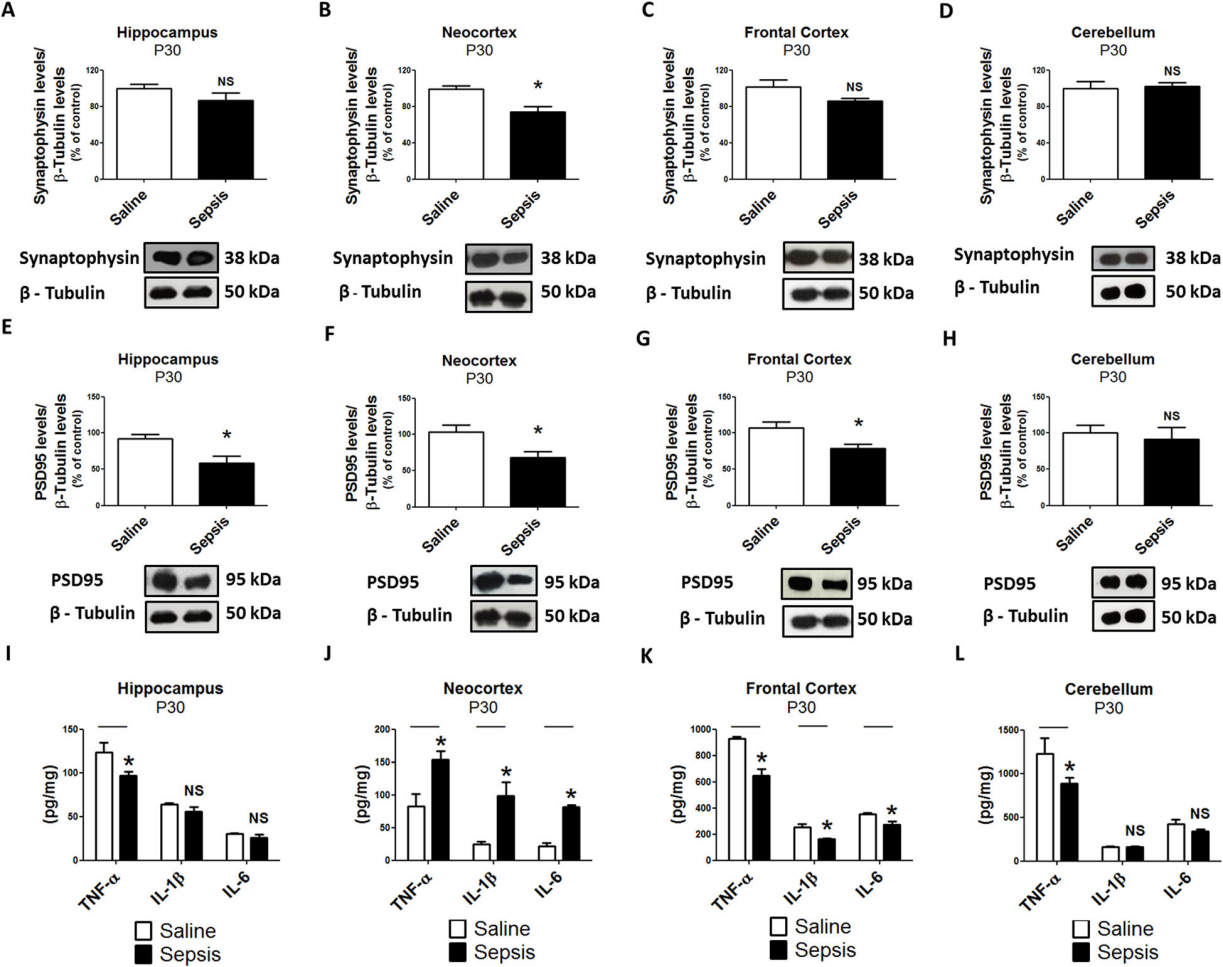
Gestational sepsis downregulates synaptophysin and PSD95 expression, and induces immune activation in young offspring brains (P30). **a**–**d** Western blot was performed to detect synaptophysin and β-tubulin in the hippocampus (**a**), neocortex (**b**), frontal cortex (**c**), and cerebellum (**d**). Each bar is the mean +/− SEM from 3 to 7 animals. **e**–**h** Western blot was performed to detect PSD95 and β-tubulin in the hippocampus (**e**), neocortex (**f**), frontal cortex (**g**), and cerebellum (**h**). Each bar is the mean +/− SEM from 3 to 4 animals. **i**–**l** The immune response at P30 was evaluated through TNF-α, IL-1β, and IL-6 levels analyzed by ELISA in the hippocampus (**i**), neocortex (**j**), frontal cortex (**k**), and cerebellum (**l**). Each bar is the mean +/− SEM from 3 animals from different litter each one. *p < 0.05 compared saline to sepsis. *NS* not significant

**Fig. 6:**
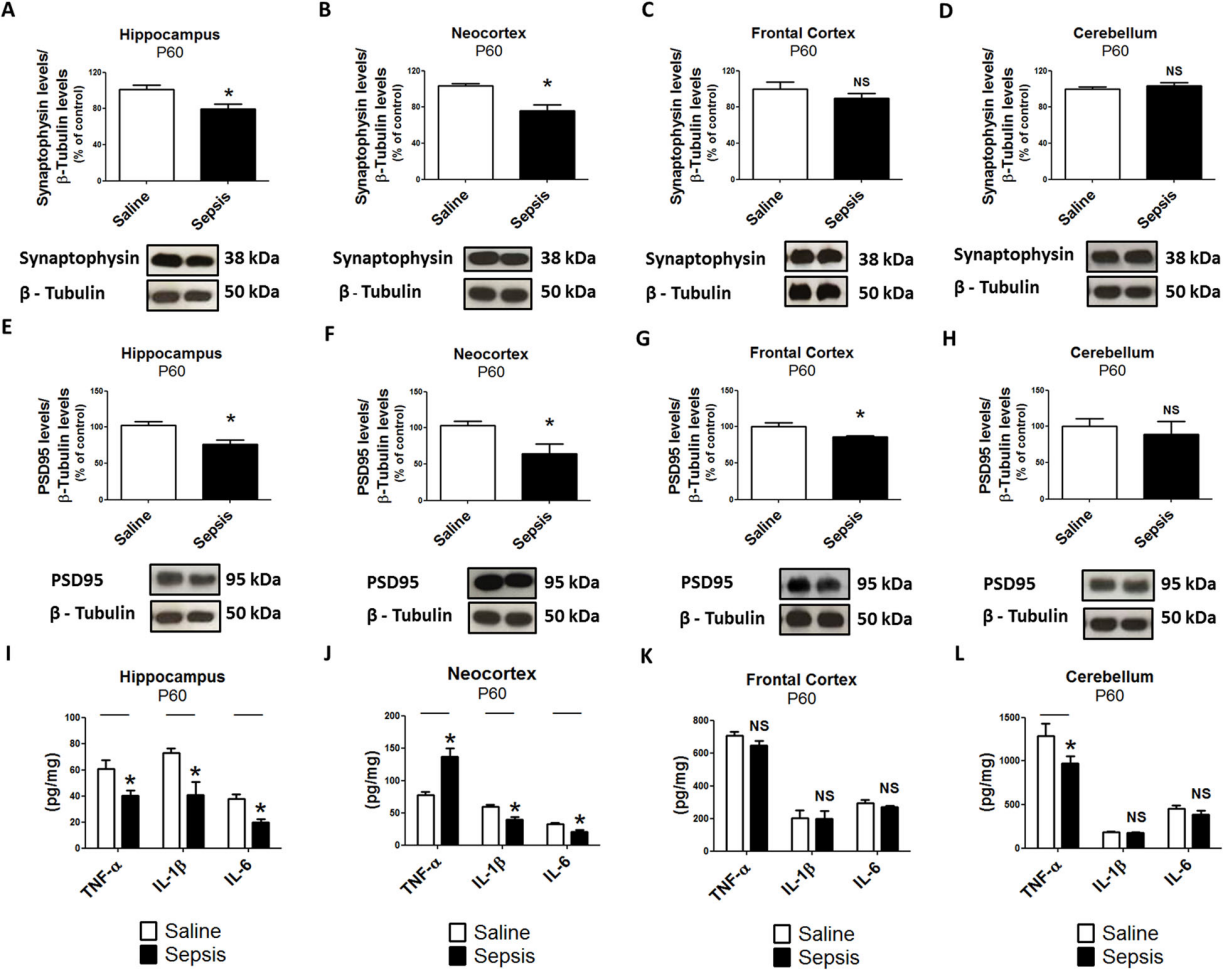
Gestational sepsis downregulates synaptophysin and PSD95 expression and induces immune activation in adult offspring brains (P60). **a**–**d** Western blot was performed to detect synaptophysin and β-tubulin in the hippocampus (**a**), neocortex (**b**), frontal cortex (**c**), and cerebellum (**d**). Each bar is the mean +/− SEM from 4 to 7 animals. **e**–**h** Western blot was performed to detect PSD95 and β-tubulin in the hippocampus (**e**), neocortex (**f**), frontal cortex (**g**), and cerebellum (**h**). Each bar is the mean +/− SEM from 3 to 4 animals. **i**–**l** The immune response at P60 was evaluated through TNF-α, IL-1β, and IL-6 levels analyzed by ELISA in the hippocampus (**i**), neocortex (**j**), frontal cortex (**k**), and cerebellum (**l**). Each bar is the mean +/− SEM from 5 to 9 animals. *p < 0.05 compared saline to sepsis. *NS* not significant

We next analyzed the biological profile of young (P30) and adult (P60) offspring to determine if the impact of gestational sepsis persists in later periods of development. TNF-α, IL-1β, and IL-6 levels were still increased in the brain in young mice, but, surprisingly, not in the liver or lung (Additional File 4A, 4B, and 4C). In adult mice, TNF-α was still elevated in the brain, but IL-1β and IL-6 had fallen to below control levels (Additional File 4D, 4E, and 4F). When we specifically examined the neocortex and the hippocampus, synaptophysin and PSD95 were consistently decreased in those areas in both young and adult mice (Figs. 5a, b, e, f and 6a, b, e, f). Pro-inflammatory cytokines were increased in the neocortex of young mice but only TNF-α remained elevated in the neocortex in adult mice (Figs. 5j and 6j). In the hippocampus, interestingly, TNF-α was decreased in young mice. In adult mice, TNF-α, IL-1β, and IL-6 levels were all decreased (Figs. 5i and 6i). We did not observe statistically significant changes of synaptic proteins from frontal cortex and cerebellum in young and adult ages, except for PSD95, which was significantly reduced in frontal cortex from adults (Figs. 5c, d, g, h and 6c, d, g). In the frontal cortex of animals at P30, the levels of cytokines were decreased in comparison to samples from the control group, but this pattern was not maintained at P60 (Figs. 5k and 6k). TNF-α was the only cytokine downregulated in the cerebellum in both young and adult mice (Figs. 5l and 6l).

### Gestational sepsis has long-term impacts on learning and memory of the offspring

To investigate if the neuroinflammatory and synaptic alterations observed in the offspring would correlate with behavioral changes at later stages of development, we employed a set of well-established behavioral tests to investigate learning abilities and memory in young and adult mice. First, we used the elevated plus maze test and the light/dark box test, which demonstrated that mice born from mothers with gestational sepsis did not exhibit greater signs of anxiety behavior (Additional File 5).

**Fig. 7:**
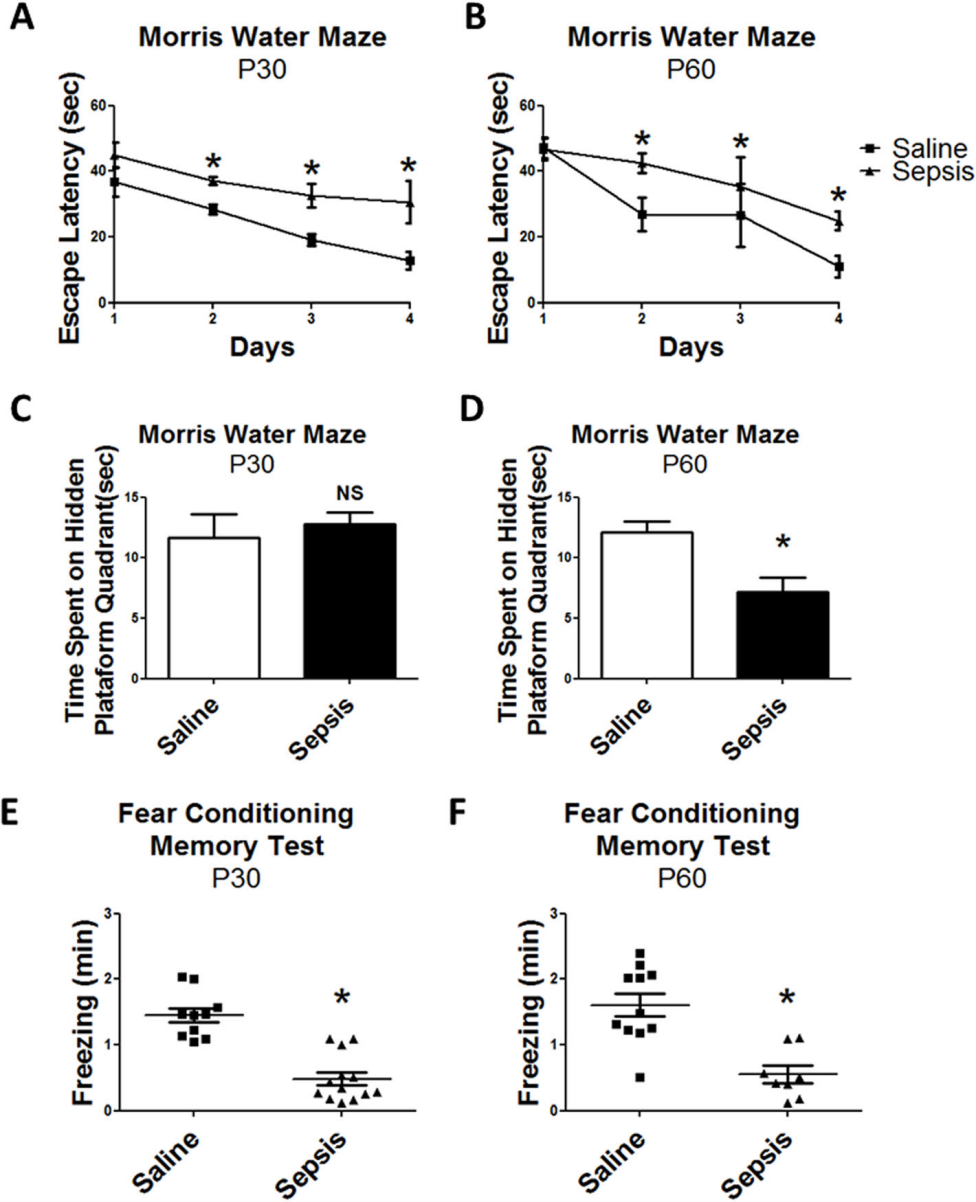
Cognitive impairment of young (P30) and adult (P60) offspring from mice subjected to gestational sepsis. **a**–**d** Morris water maze test. **a**, **b** The amount of time spent by young (**a**) and adult (**b**) offspring to climb the hidden platform along 4 days of experiment was measured. Each point is the mean +/− SEM from 9 to 12 animals. **c**, **d** Evaluated the time on the hidden platform quadrant of young (**c**) and adult (**d**) offspring. Each bar is the mean +/− SEM from 9 to 12 animals. **e**, **f** Aversive memory was evaluated in the fear conditioning test at young **e**) and adult (**f**) ages evaluating the time spent in freezing position. Each group of saline or sepsis administration is represented by a square or triangle, and the mean +/− SEM from 9 to 12 animals are indicated by lines. *p < 0.05 compared saline to sepsis. *NS* not significant

Our next step was to apply the Morris water maze test to compare learning and spatial memory between both groups. Offspring of both ages (P30 and P60) from septic mice took longer to find the hidden platform during the course of the 4 days of the experiment in comparison to offspring from the control group (Fig. 7a, b). When we tested for integrity of spatial memory, young mice born from mothers with gestational sepsis did not show any signs of impairment (Fig. 7c). However, adult mice spent less time in the hidden platform quadrant, suggesting impairment in spatial memory at later stages of development (Fig. 7d). We next evaluated aversive memory using this fear conditioning memory test. Young and adult offspring from septic mice spent less time in the freezing position than the offspring from the control group (Fig. 7e, f), indicating possible impairments in aversive memory circuitry.

**Fig. 8:**
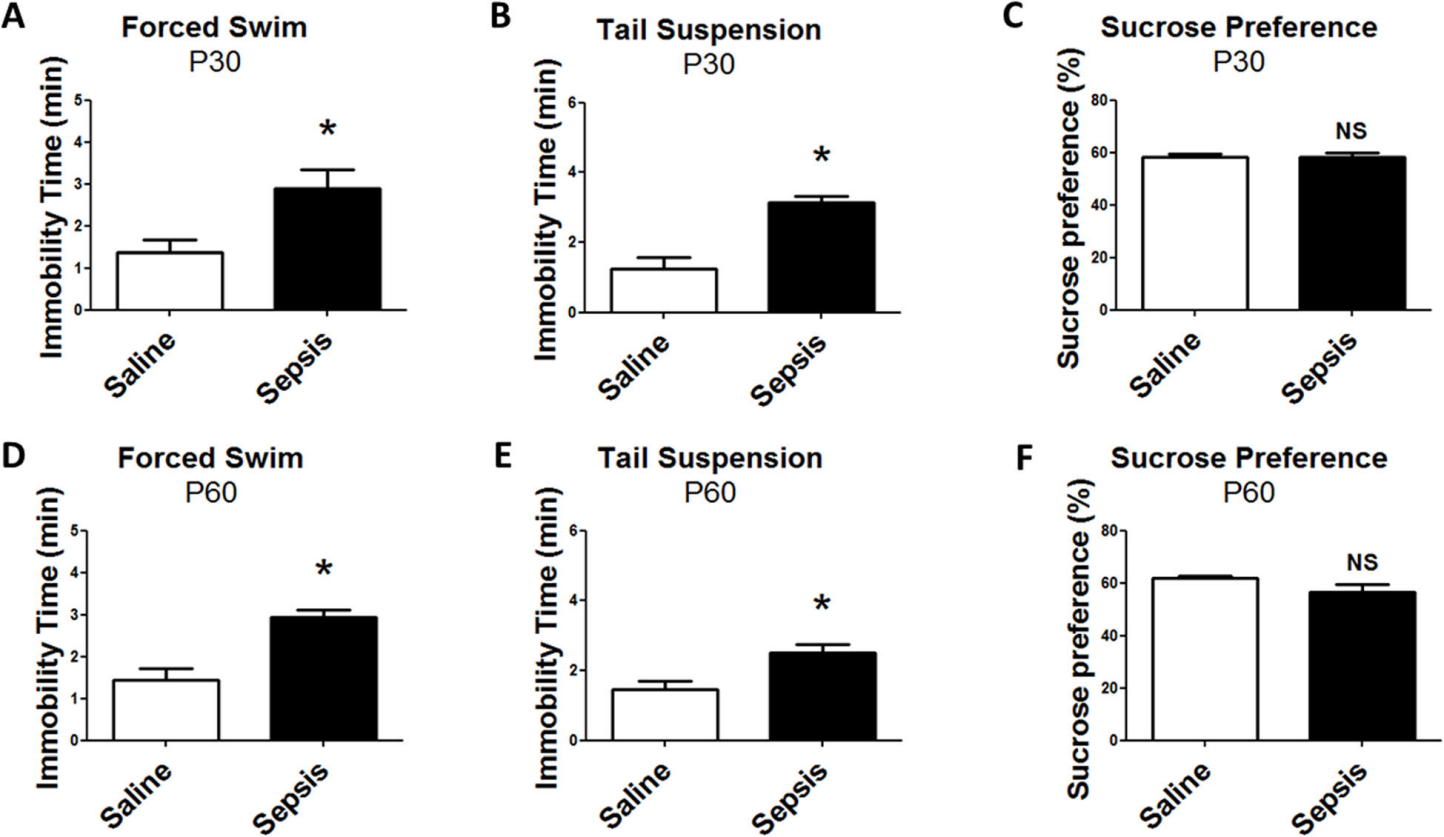
Depressive behavior of offspring from mice subjected to gestational sepsis. The forced swim test (**a**, **d**) and tail suspension test (**b**, **e**) evaluated depressive-like behavior of young (**a**–**c**) and adult (**d**, **e**) animals. Anhedonia was tested by the sucrose preference test (**c**, **f**). Each bar is the mean +/− SEM from 9 to 12 animals. *p < 0.05 compared saline to sepsis. *NS* not significant

We also tested depressive-like behavior using the forced swim, tail suspension, and sucrose preference tests. We found that young and adult offspring from septic mice had higher immobility time than offspring from the control group in the forced swim test (Fig. 8a, d). Corroborating this result, the immobility times in the tail suspension test were higher in offspring from the sepsis group than the control group (Fig. 8b, e). This suggests that gestational sepsis may contribute to the development of depressive-like behavior in adulthood. The sucrose preference test was used to evaluate the degree of anhedonia, a symptom of depressive disorders. However, there was no significant difference in sucrose preference among offspring from sepsis and control groups in young and adult mice (Fig. 8c, f), suggesting that the animals did not show signs of anhedonia and the system of reward processing was not affected by gestational sepsis.

## Discussion

Here, we describe a model of gestational sepsis induced by intratracheal instillation of 3 × 10^8^ *Klebsiella pneumoniae*. This Gram-negative bacterium represents one of the most important causes of neonatal sepsis, resulting in a high mortality rate in humans [33]. To validate this model, we developed a clinical score that was used to identify and grade severity of sepsis in pregnant mice, confirming the success of bacteria instillation. Also, it allowed us to implement antibiotic therapy in order to rescue the pregnant mice from sepsis during the completion of the gestational period. This protocol enabled us to study short- and long-term consequences of sepsis in the offspring. To our knowledge, this is the first model of gestational pneumosepsis in mice that allows the study of short- and long-term consequences in the offspring and that unveils previously unrecognized consequences of sepsis at different stages of development.

Sepsis causes 11% of maternal deaths worldwide. The incidence is the highest in Southern Asia and Sub-Saharan Africa, although the burden is probably underestimated because of the lack of uniform definition for maternal sepsis [34, 35]. It has been reported in humans that many pathological conditions in the mother can adversely impact neonatal life as well as later stages of development [36–39]. Moreover, accumulating experimental evidence demonstrates that maternal immune activation leads to behavioral changes in adult offspring, which are related to changes in dopaminergic and glutamatergic systems, hypothalamic–pituitary–adrenal (HPA) axis activation, and pro- and anti-inflammatory cytokine profiles [40–44]. For the most part, these preclinical investigations used surrogate models of prenatal bacterial infection such as administration of LPS [45]; turpentine [43]; or prenatal viral-like infection by administration of polyriboinosinic–polyribocytidilic acid (Poly I:C) [44] to experimental animals such as rats and guinea pigs. Our observations are built upon this existing literature. Using a clinically relevant model of infection of pregnant mice with *Klebsiella pneumoniae*, we observed that offspring from septic mice showed reduced size and weight during the neonatal period. This suggests an impaired development that could be related to intrauterine infectious and inflammatory alterations as detected by elevated cytokine levels in the placenta at embryonic day 15 (E15). Inflammatory alterations were still present in the liver, lung, and brain of neonates at P2. Moreover, TNF-α levels remained elevated even at P8, indicating that the effect of infectious inflammation lasted for a long time and may continue to disrupt the behavior of the offspring, affecting the function of different systems including the CNS. Prenatal exposure to inflammatory cytokines such as TNF-α, IL-1β, and IL-6 has been linked to neurodevelopmental alterations in the offspring. Samuelson et al. [46] showed that adult rats subjected to pre-natal IL-6 treatment displayed a marked increase in hippocampal astrogliosis and apoptosis, together with an enhanced expression of the GABAA α5- and *N*-methyl-d-aspartate (NMDA) receptor NR1-subunits. Moreover, prenatal exposure to IL-6 also resulted in the deficits of spatial learning deficit in the Morris water maze [46].

Inflammatory cytokines, including IL-1β, TNF-α, and IL-6, are produced in the CNS and are involved in the regulation of excitability of neurons [47]. However, elevated levels of cytokines in neuroinflammatory processes also result in neuronal dysfunctions, such as deregulated synaptic transmission and neuronal excitability [48].

In neocortical and hippocampal neurons, TNF-α and IL-1β seem to play important roles in synaptic plasticity and excitotoxicity [47, 49, 50]. Both cytokines are associated with long-term potentiation (LTP) in the hippocampus through glutamate ionotropic receptors, α-amino-3-hydroxy-5-methyl-4-isoxazolepropionic acid (AMPA) receptors, and NMDA receptor activation [47, 51]. Also, TNF-α and IL-1β are associated with a reduction in synaptophysin, suggesting that high levels of both cytokines, as found in neuroinflammation, results in loss of synapses [52, 53]. In addition, IL-1β also promotes the GABAergic inhibition through enhanced calcium influx in the hippocampus [54]. Han et al. [55] studied cerebral cortex from septic mice and found that microglia-derived IL-1β may disturb axon development and synapse formation with significant neuronal loss. In that study, the synaptic deficit was proven by reduction of synaptophysin expression through a p38-MAPK signaling pathway. Also, in the hippocampus of IL-6 transgenic mice, chronic exposure to IL-6 inhibited neurogenesis [56]. Conroy et al. [57] observed that neuronal cluster size was reduced in cerebellar granule culture treated with IL-6, suggesting that IL-6 exposure results in neuronal loss. In the same study, synaptic proteins (synapsin I and II), enolase, and α*-*internexin were significantly decreased after IL-6 treatment. They demonstrated that IL-6 induces toxicity to the granule neurons and that it can be even more damaging when combined with excitotoxic conditions involving excessive glutamate and activation of NMDAR as observed in IL-1β and TNF*-*α stimulation [58]. Further, in vivo application of LPS and heat killed Gram-negative bacteria induced the increase in the intrinsic excitability of cerebellar Purkinje cells and synaptic transmission, which were associated with both depressive- and autistic-like behaviors [59–61].

We observed that TNF-α and IL-1β were elevated at P2 and that IL-6 was still elevated in the brains of neonates at P8. This increase in pro-inflammatory cytokines in neonates from gestational sepsis is likely to be related to intrauterine pro-inflammatory stimuli, resulting in an unbalanced neuroimmune response during the development that induced behavioral changes in adult offspring as a final outcome of the abnormal neural development. These long-lasting effects are also indicated by alterations in cytokine levels detected even in adult life. Although no other intentional stimulus was given to the offspring, cytokine levels remained elevated in the brains for 30 days and longer. In particular, TNF-α was still elevated in the brains of adult mice (P60).

While variation in specific pathogens during gestation appears to be a minor factor on long-term behavioral alterations in offspring [62], the precise time at when infection occurs during gestation plays a major role in both structural and behavioral postnatal alterations [62, 63]. We chose to induce sepsis at E14. This corresponds to Carnegie stage 19 in mouse gestation and approximately 9 weeks of gestational age in humans [64].

In fact, developmental events leading to the establishment of the central dopaminergic system in both rodents and humans start early in fetal life, and therefore, the neural circuit may be vulnerable to infectious challenges during this gestational period. This could be the basis for certain behavioral dysfunctions in later life, including motor deficits, learning and memory impairments, and depression [62]. Motor deficits secondary to sepsis have been described in patients admitted to the ICU [65]. Moreover, intracervical inoculation of LPS in pregnant mice induced a sensory-motor impairment and deficit of reflex and motor skills in the offspring [66]. Our results corroborate this positivity, since offspring from septic mice showed both motor deficits during the early and late neonatal period and impairments of learning and memory at older ages, as we observed significant alterations in all motor tests in sepsis group compared to control group, except for the negative geotaxis test at P2. These tests may indicate deficits in balance, coordination, or vestibular input. The negative geotaxis test also requires motor and vestibular input for the mouse to recognize its orientation and rotate, but, the average age for the negative geotaxis reflex to be observed in rodents is the 7th day of life, ranging from the 3rd to the 15th day of life [67]. Thus, neonatal motor impairments represent deficits in neuromotor reflexes at early and late neonatal periods and may explain some of our findings. In another study, however, the neonatal mouse model of cerebral palsy at 7 and 13 days of age showed no significant differences in negative geotaxis and cliff aversion tests when affected animals were compared to the control group [17], demonstrating that these deficits could also be preserved in a later neonatal stage, needing further investigation on what could be causing the different results.

In order to evaluate neuromuscular function and hindlimb strength, neonatal mice were submitted to hindlimb suspension test [17]. Neonatal animals from the sepsis group demonstrated more time hanging at the tube compared to the control groups, showing a reduction of mobility when exposed to stressful situations. They also assumed a depressive-like behavior similar to young and adult mice when submitted to tail suspension and forced swim tests.

It is known from clinical studies that neuropsychiatric problems, including depression and anxiety, occur in sepsis survivors [68]. Also, sepsis-associated encephalopathy is associated with depressive symptoms in survivors [69]. Likewise, pre-clinical studies using the cecal ligation and puncture model of sepsis in neonates demonstrated that animals that showed neonatal sepsis exhibited depressive-like behavior in adulthood [20]. Similarly, our findings show that offspring from septic mothers exhibit patterns consistent with depressive-like behavior. This fact suggests that gestational sepsis could predisposes to depression-like behavior outcomes in both young and adult offspring. Furthermore, gestational sepsis causes less adversative and escape instincts in the offspring, as they remain immobile for longer than the control group but without presenting anhedonia. We performed the elevated plus maze test to examine anxiety-like behavior, but only the total number of entries from offspring at P30 was significantly higher in the sepsis group compared to control offspring. Therefore, in order to further investigate if the offspring of mothers with gestational sepsis display anxious behavior, we next performed the light/dark box test, but there was no difference between the groups, leading us to conclude that offspring from septic mice do not display this behavior pattern.

In the present work, we demonstrated that induction of gestational sepsis promotes not only immune alterations but also long-term adverse neuropsychiatric-like outcomings in the offspring. Although behavioral and cognitive alterations have been described in the offspring after maternal challenge with pro-inflammatory stimuli, detailed cellular and molecular mechanisms associated with these alterations are still lacking. It is known that synaptophysin protein modulates the efficiency of synaptic vesicle cycles. Disturbance in synaptophysin expression has been associated with synaptic deficits as observed in Alzheimer’s disease [70]. Also, deletion of synaptophysin is associated with impaired object recognition and reduced spatial learning [71]. PSD95 is an essential postsynaptic protein that plays an important role in synaptic plasticity and neurodevelopment via glutamatergic synapses. PSD95 dysfunction has been associated with neuropsychiatric disorders, including schizophrenia, autism and Alzheimer’s disease [72–74].

Calcium/calmodulin-dependent protein kinase II (CaMKII) is one of the most abundant brain proteins and is a well-known regulator of synaptic events underlying plasticity, learning, and cognition. CaMKII activity has also been shown to be essential to neuronal survival and function to long-term potentiation [75]. It might, therefore, be useful to assess CaMKII levels in different brain regions of the offspring from septic animals. In fact, Turlova et al. [76] demonstrated a decrease in CaMKII expression 6 and 24 h after ischemic brain injury in neonates that was associated with motor and cognition deficits in 8 day old and 35-day-old animals.

Recently, a decrease in synaptophysin and PSD95 expression was described following neuroinflammation induced by the cecal ligation and puncture (CLP) model of sepsis [77, 78] or after intraperitoneally administration of LPS—a component of Gram-negative bacteria [79–82]. There are few studies that correlate infection during gestation and disturbance of synaptophysin expression in the offspring and none using bacterial sepsis models. A study that relates prenatal immune activation and hippocampal deficits in the offspring induced by the viral mimetic Poly I:C showed a reduction of synaptophysin relative to controls. However, quantitative analyses of inflammatory cytokines in hippocampus and plasma showed no persistent inflammation in the offspring [83]. Similarly, we found that synaptophysin and PSD95 levels were lower in animals born from mothers with gestational sepsis. This was demonstrated especially in the hippocampus and neocortex from neonates and persisting into adult phase. Nevertheless, IL-1β and IL-6 were elevated in the hippocampus at P2 and P30 and in the neocortex at P8 and P30 in mice from the sepsis group. The apparent discrepancies between cytokine profile may be explained by the magnitude or nature of the initial challenge. While Giovanoli et al. [83] used a viral mimetic substance, we used a live pathogen to induce bacterial sepsis that was subsequently treated. This resulted in septic physiology that could be monitored by a clinical score that enabled us to assess the severity of the condition. Despite the antibiotic treatment, several clinical signs (Fig. 1) were still recorded in the sepsis group, reflecting the severity of the initial challenge. This may account for the different cytokine profiles detected in our experiments, but further investigations are needed for confirmation. Another study analyzed the fetal brain 6 h, 12 h, and 24 h after LPS instillation in E15 Wistar rats and reported increased concentrations of IL-1β, TNF-α, and IL-6 compared to the control group [84]. This may also indicate that the nature of the initial source of infection to the mother induces different neural cytokine responses in the neonate.

It is well described that in the adult population, immune response patterns evolve toward an immunosuppressive phenotype after sepsis [85]. This is clinically associated with a high incidence of re-hospitalizations, secondary infections, and other immune dysregulation as patients recover from sepsis [86]. Our data show a decrease in basal cytokines levels in adult offspring that may reflect an immunosuppressive phenotype. Whether or not these animals present a suppressed response and increased susceptibility to an infection remains to be addressed in future investigations.

Our work was limited by a few challenges that we hope to be able to overcome in the future as we continue our investigation in this relevant topic. First, we did not use females in our behavioral analyses because the variability in the response would require an increased number of animals that would demand for animal housing and also increase the costs, making the study impracticable. Second, we did not discuss CaMKII activity in the present study since we were not yet able to assess it in our experiments. CaMKII may have important roles in learning and cognition and measuring its expression would be informative to further describe the mechanistic aspects about the impact of gestational sepsis upon the offspring. Third, we did not perform synaptic or cytokine analysis in sub-regions of the cortex and the hippocampus that would help make correlations with the behavior phenotype.

## Conclusion

Our results suggest that gestational sepsis induces an inflammatory response in neonatal mice, negatively impacts CNS development, and leads to cognitive and behavioral alterations in adult offspring. Each of these alterations can be linked to synaptic damage, suggesting that maternal sepsis is a neglected risk factor for depression, learning and memory impairments in childhood and adult life.

## Supplementary Information

**Additional file 1.** Additional table 1 Statistical analyses.

**Additional file 2.** Additional table 2 Clinical conditions of Gestational Sepsis.

**Additional file 3.** Pre-natal exposure to sepsis induces neonatal systemic inflammatory response. (A-C) TNF-α, IL-1β and IL-6 levels analyzed by ELISA in the liver (A), lungs (B) and brain (C) P2. Each bar is the mean +/- SEM from at least 5 animals. (D-F) TNF-α, IL-1β and IL-6 levels analyzed by ELISA in the liver (D), lungs (E) and brain (F) P8. Each bar is the mean +/- SEM from at least 5 animals. * p<0.05 comparing saline to sepsis.

**Additional file 4.** Pre-natal exposure to sepsis impacts in systemic inflammatory response in young and adult offspring. (A-C) TNF-α, IL-1β and IL-6 levels analyzed by ELISA in the liver (A), lungs (B) and brain (C) P30. Each bar is the mean +/- SEM from at least 5 animals. (D-F) TNF-α, IL-1β and IL-6 levels analyzed by ELISA in the liver (D), lungs (E) and brain (F) P60. Each bar is the mean +/- SEM from at least 5 animals. * p<0.05 comparing saline to sepsis.

**Additional file 5.** Effects of gestational sepsis in young and adult offspring anxiety-like behavior of P30 and P60. (A-B) The elevated plus maze data were measured and compared of P30 offspring by the time spent in open arms (A) and total number of entries into open arms (B). Each point is the mean +/- SEM n= 9-12. (C-D) Elevated plus maze of P60 offspring analyzed by the time spent in open arms (C) and total number of entries into open arms (D). Each point is the mean +/- SEM n= 9-12. (E-J) The light/dark box test exposed the P30 offspring (E-J) and P60 (H-J) offspring to an aversive ambient (lit compartment) and evaluate the time spent on lit compartment (E, H), number of entrances on the lit compartment (F, I), and time spent in transitions between the lit and dark compartments (G, J). Each bar is the mean +/- SEM from n= 9-12 * p<0.05 comparing saline to sepsis.

## Data Availability

The datasets used and analyzed during the current study are available from the corresponding author on reasonable request.

## Abbreviations

ASD: Autism spectrum disorder
CNS: Central nervous system
ICU: Intensive care units
IL: Interleukin
LPS: Lipopolysaccharide
MAC: Minimum alveolar concentration
SAE: Sepsis-associated encephalopathy
SEM: Standard error of the mean
SW: Swiss Webster
TNF: Tumor necrosis factor
WHO: World Health Organization
CFU: Colony-forming unit
S.C: Subcutaneously
WB: Western Blot
ELISA: Enzyme-linked immunosorbent assay
P: Postnatal day
E: Embryonic day
FIOCRUZ: Oswaldo Cruz Foundation
PBS: Phosphate-buffered saline
RPM: Rotations per minute
HPA: Hypothalamic–pituitary–adrenal
Poly I:C: Polyriboinosinic–polyribocytidilic acid
NMDA: *N*-methyl-d-aspartate
AMPA: α-Amino-3-hydroxy-5-methyl-4-isoxazolepropionic acid
