# Supplementary material for: Inflammatory, synaptic, motor, and behavioral alterations induced by gestational sepsis on the offspring at different stages of life
Source: J Neuroinflammation. 2021 Feb 25;18:60. doi: 10.1186/s12974-021-02106-1 (PMC7905683; doi:10.1186/s12974-021-02106-1)
Supplement: Supplementary file 1 — Additional file 1. Additional table 1 Statistical analyses. [file 12974_2021_2106_MOESM1_ESM.docx]

| **Additional table 1 - Statistical analyses.** | | |  |  |  |  |
| --- | --- | --- | --- | --- | --- | --- |
| **Figure 1A.** Clinical Score of pregnant mice | | |  |  |  |  |
| Hours | Difference | t | P value | Summary | t=2.424 |  |
| 0 | 0.0000 | 0.0000 | P > 0.05 | NS | df=10 |  |
| 5 | 1.950 | 2.750 | P < 0.05 | * | Qui-square= 0.466 | |
| 24 | 3.950 | 6.824 | P<0.001 | *** |  |  |
| 48 | 2.783 | 4.301 | P<0.001 | *** |  |  |
| 72 | 1.858 | 2.871 | P < 0.05 | * |  |  |
| 96 | 0.4750 | 0.7339 | P > 0.05 | NS |  |  |
|  |  |  |  |  |  |  |
| **Figure 1B.** Clinical Score of pregnant mice treated with LPS | | | |  |  |  |
| Saline vs 3 mg/kg |  |  |  |  |  |  |
| Hour | Difference | t | P value | Summary |  |  |
| 0 | 0.0000 | 0.0000 | P > 0.05 | NS |  |  |
| 5 | 1.000 | 1.279 | P > 0.05 | NS |  |  |
| 24 | 2.900 | 3.710 | P<0.01 | ** |  |  |
| 48 | 0.9000 | 1.151 | P > 0.05 | NS |  |  |
| Saline vs 10 mg/kg |  |  |  |  |  |  |
| Hour | Difference | t | P value | Summary |  |  |
| 0 | 0.0000 | 0.0000 | P > 0.05 | NS |  |  |
| 5 | 4.000 | 5.117 | P<0.001 | *** |  |  |
| 24 | 4.233 | 5.415 | P<0.001 | *** |  |  |
| 48 | 2.900 | 3.710 | P<0.01 | ** |  |  |
| Saline vs 15 mg/kg |  |  |  |  |  |  |
| Hour | Difference | t | P value | Summary |  |  |
| 0 | 0.0000 | 0.0000 | P > 0.05 | NS |  |  |
| 5 | 3.667 | 4.690 | P<0.001 | *** |  |  |
| 24 | 5.567 | 7.121 | P<0.001 | *** |  |  |
| 48 | 5.900 | 7.547 | P<0.001 | *** |  |  |
|  |  |  |  |  |  |  |
| Source of Variation | Df | Sum-of-squares | Mean square | F |  |  |
| Interaction | 9 | 46.47 | 5.163 | 6.638 |  |  |
| Time | 3 | 68.66 | 22.89 | 29.42 |  |  |
| Column Factor | 3 | 101.0 | 33.68 | 25.26 |  |  |
| Subjects (matching) | 8 | 10.67 | 1.333 | 1.714 |  |  |
| Residual | 24 | 18.67 | 0.7778 |  |  |  |
|  |  |  |  |  |  |  |
|  |  |  |  |  |  |  |
| **Figure 1C.** Placenta - E15 | |  |  |  |  | Summary |
| Mann Whitney test | P value | U | n | Unpaired t test | P value |  |
| TNF-alpha - Saline vs Sepsis | |  |  |  |  |  |
|  | 0.05 | 0 | 12 |  | 0.041 | * |
| IL-1 beta - Saline vs Sepsis | |  |  |  |  |  |
|  | 0.05 | 0 | 12 |  | 0,0088 | * |
| IL-6 - Saline vs Sepsis | |  |  |  |  |  |
|  | 0.05 | 0 | 12 |  | 0.0418 | * |
|  |  |  |  |  |  |  |
| **Figure 1E.** Offspring weight of P2 | | |  |  |  |  |
| Mann Whitney test | P value | U | n | Unpaired t test | P value |  |
| Saline vs Sepsis | 0.0003 | 0 | 20 |  | 0.0001 | * |
|  |  |  |  |  |  |  |
| **Figure 1F.** Offspring weight of P8 | | |  |  |  |  |
| Mann Whitney test | P value | U | n | Unpaired t test | P value |  |
| Saline vs Sepsis | 0.0008 | 10 | 20 |  | 0.0005 | * |
|  |  |  |  |  |  |  |
| **Figure 2A.** Cliff Aversion test - P2 | |  |  |  |  |  |
| Mann Whitney test | P value | U | n | Unpaired t test | P value |  |
| Saline vs Sepsis | 0.05 | 0 | 3 |  | 0.0039 | * |
|  |  |  |  |  |  |  |
| **Figure 2B.** Geotaxis test - P2 | |  |  |  |  |  |
| Mann Whitney test | P value | U | n | Unpaired t test | P value |  |
| Saline vs Sepsis | 0.2 | 2 | 3 |  | 0.2638 | NS |
|  |  |  |  |  |  |  |
| **Figure 2C.** Hindlimb Suspension test - P2 | | |  |  |  |  |
| Mann Whitney test | P value | U | n | Unpaired t test | P value |  |
| Saline vs Sepsis | 0.05 | 0 | 3 |  | 0.0128 | * |
|  |  |  |  |  |  |  |
| **Figure 2D.** Cliff Aversion test - P8 | | |  |  |  |  |
| Mann Whitney test | P value | U | n | Unpaired t test | P value |  |
| Saline vs Sepsis | 0.05 | 0 | 3 |  | 0.0234 | * |
|  |  |  |  |  |  |  |
| **Figure 2E.** Geotaxis test - P8 | |  |  |  |  |  |
| Mann Whitney test | P value | U | n | Unpaired t test | P value |  |
| Saline vs Sepsis | 0.05 | 0 | 3 |  | 0.0205 | * |
|  |  |  |  |  |  |  |
| **Figure 2F.** Hindlimb Suspension test - P8 | | |  |  |  |  |
| Mann Whitney test | P value | U | n | Unpaired t test | P value |  |
| Saline vs Sepsis | 0.05 | 0 | 3 |  | 0.0199 | * |
|  |  |  |  |  |  |  |
| **Figure 2G.** Rota Rod - P30 | |  |  |  |  |  |
| Mann Whitney test | P value | U | n | Unpaired t test | P value |  |
| Saline vs Sepsis | 0.4124 | 4.5 | 3 |  | 0.4903 | NS |
|  |  |  |  |  |  |  |
| **Figure 2H.** Rota Rod - P60 | |  |  |  |  |  |
| Mann Whitney test | P value | U | n | Unpaired t test | P value |  |
| Saline vs Sepsis | 0.4124 | 3.5 | 3 |  | 0.3859 | NS |
|  |  |  |  |  |  |  |
| **Figure 3A.** Synaptophysin Levels - Hippocampus – P2 | | | |  |  |  |
| Mann Whitney test | P value | U | n | Unpaired t test | P value |  |
| Saline vs Sepsis | 0.0087 | 6 | 7 |  | 0.0041 | * |
|  |  |  |  |  |  |  |
| **Figure 3B.** Synaptophysin Levels - Neocortex – P2 | | |  |  |  |  |
| Mann Whitney test | P value | U | n | Unpaired t test | P value |  |
| Saline vs Sepsis | 0.0152 | 3 | 6 |  | 0.0059 | * |
|  |  |  |  |  |  |  |
| **Figure 3C.** Synaptophysin Levels - Frontal Cortex – P2 | | | |  |  |  |
| Mann Whitney test | P value | U | n | Unpaired t test | P value |  |
| Saline vs Sepsis | 0.2533 | 2.5 | 3 |  | 0.3422 | NS |
|  |  |  |  |  |  |  |
| **Figure 3D.** Synaptophysin Levels - Cerebellum – P2 | | | |  |  |  |
| Mann Whitney test | P value | U | n | Unpaired t test | P value |  |
| Saline vs Sepsis | 0.0286 | 0 | 4 |  | 0.0003 | * |
|  |  |  |  |  |  |  |
| **Figure 3E.** PSD95 Levels - Hippocampus – P2 | | |  |  |  |  |
| Mann Whitney test | P value | U | n | Unpaired t test | P value |  |
| Saline vs Sepsis | 0.0143 | 0 | 4 |  | 0.0214 | * |
|  |  |  |  |  |  |  |
| **Figure 3F.** PSD95 Levels - Neocortex – P2 | | |  |  |  |  |
| Mann Whitney test | P value | U | n | Unpaired t test | P value |  |
| Saline vs Sepsis | 0.2 | 2 | 3 |  | 0.1847 | NS |
|  |  |  |  |  |  |  |
| **Figure 3G.** PSD95 Levels - Frontal Cortex – P2 | | |  |  |  |  |
| Mann Whitney test | P value | U | n | Unpaired t test | P value |  |
| Saline vs Sepsis | 0.05 | 0 | 3 |  | 0.0615 | * |
|  |  |  |  |  |  |  |
| **Figure 3H.** PSD95 Levels - Cerebellum – P2 | | |  |  |  |  |
| Mann Whitney test | P value | U | n | Unpaired t test | P value |  |
| Saline vs Sepsis | 0.0143 | 0 | 3 |  | 0.0081 | * |
|  |  |  |  |  |  |  |
| **Figure 3I.** Hippocampus – P2 | |  |  |  |  |  |
| Mann Whitney test | P value | U | n | Unpaired t test | P value |  |
| TNF-alpha - Saline vs Sepsis | |  |  |  |  |  |
|  | 0.05 | 0 | 3 |  | 0.0027 | * |
| IL-1 beta - Saline vs Sepsis | |  |  |  |  |  |
|  | 0.05 | 0 | 3 |  | 0.0038 | * |
| IL-6 - Saline vs Sepsis | |  |  |  |  |  |
|  | 0.4 | 2 | 3 |  | 0.1407 | NS |
|  |  |  |  |  |  |  |
| **Figure 3J.** Neocortex – P2 | |  |  |  |  |  |
| Mann Whitney test | P value | U | n | Unpaired t test | P value |  |
| TNF-alpha - Saline vs Sepsis | |  |  |  |  |  |
|  | 0.05 | 0 | 3 |  | 0.0006 | * |
| IL-1 beta - Saline vs Sepsis | |  |  |  |  |  |
|  | 0.05 | 0 | 3 |  | 0.014 | * |
| IL-6 - Saline vs Sepsis | |  |  |  |  |  |
|  | 0.35 | 3 | 3 |  | 0.3223 | NS |
|  |  |  |  |  |  |  |
| **Figure 3K.** Frontal Cortex – P2 | |  |  |  |  |  |
| Mann Whitney test | P value | U | n | Unpaired t test | P value |  |
| TNF-alpha - Saline vs Sepsis | |  |  |  |  |  |
|  | 0.2 | 2 | 3 |  | 0.1681 | NS |
| IL-1 beta - Saline vs Sepsis | |  |  |  |  |  |
|  | 0.2 | 2 | 3 |  | 0.134 | NS |
| IL-6 - Saline vs Sepsis | |  |  |  |  |  |
|  | 0.2 | 2 | 3 |  | 0.1326 | NS |
|  |  |  |  |  |  |  |
| **Figure 3L.** Cerebellum – P2 | |  |  |  |  |  |
| Mann Whitney test | P value | U | n | Unpaired t test | P value |  |
| TNF-alpha - Saline vs Sepsis | |  |  |  |  |  |
|  | 0.2 | 2 | 3 |  | 0.0778 | NS |
| IL-1 beta - Saline vs Sepsis | |  |  |  |  |  |
|  | 0.3 | 3 | 3 |  | 0.2286 | NS |
| IL-6 - Saline vs Sepsis | |  |  |  |  |  |
|  | 0.35 | 3 | 3 |  | 0.1624 | NS |
|  |  |  |  |  |  |  |
| **Figure 4A.** Synaptophysin Levels - Hippocampus – P8 | | | |  |  |  |
| Mann Whitney test | P value | U | n | Unpaired t test | P value |  |
| Saline vs Sepsis | 0.0022 | 1 | 6 |  | 0.0005 | * |
|  |  |  |  |  |  |  |
| **Figure 4B.** Synaptophysin Levels - Neocortex – P8 | | |  |  |  |  |
| Mann Whitney test | P value | U | n | Unpaired t test | P value |  |
| Saline vs Sepsis | 0.0325 | 6 | 6 |  | 0.0309 | * |
|  |  |  |  |  |  |  |
| **Figure 4C.** Synaptophysin Levels - Frontal Cortex – P8 | | | |  |  |  |
| Mann Whitney test | P value | U | n | Unpaired t test | P value |  |
| Saline vs Sepsis | 0.1 | 3 | 4 |  | 0.0943 | NS |
|  |  |  |  |  |  |  |
| **Figure 4D.** Synaptophysin Levels - Cerebellum – P8 | | | |  |  |  |
| Mann Whitney test | P value | U | n | Unpaired t test | P value |  |
| Saline vs Sepsis | 0.2429 | 5 | 4 |  | 0.2739 | NS |
|  |  |  |  |  |  |  |
| **Figure 4E.** PSD95 Levels - Hippocampus – P8 | | |  |  |  |  |
| Mann Whitney test | P value | U | n | Unpaired t test | P value |  |
| Saline vs Sepsis | 0.05 | 0 | 3 |  | 0.0216 | * |
|  |  |  |  |  |  |  |
| **Figure 4F.** PSD95 Levels - Neocortex – P8 | | |  |  |  |  |
| Mann Whitney test | P value | U | n | Unpaired t test | P value |  |
| Saline vs Sepsis | 0.2 | 2 | 3 |  | 0.1835 | NS |
|  |  |  |  |  |  |  |
| **Figure 4G.** PSD95 Levels - Frontal Cortex – P8 | | |  |  |  |  |
| Mann Whitney test | P value | U | n | Unpaired t test | P value |  |
| Saline vs Sepsis | 0.4429 | 7 | 4 |  | 0.2741 | NS |
|  |  |  |  |  |  |  |
| **Figure 4H.** PSD95 Levels - Cerebellum – P8 | | |  |  |  |  |
| Mann Whitney test | P value | U | n | Unpaired t test | P value |  |
| Saline vs Sepsis | 0.35 | 3 | 3 |  | 0.3644 | NS |
|  |  |  |  |  |  |  |
| **Figure 4I.** Hippocampus – P8 | |  |  |  |  |  |
| Mann Whitney test | P value | U | n | Unpaired t test | P value |  |
| TNF-alpha - Saline vs Sepsis | |  |  |  |  |  |
|  | 0.4 | 2 | 3 |  | 0.1155 | NS |
| IL-1 beta - Saline vs Sepsis | |  |  |  |  |  |
|  | 0.4 | 2 | 3 |  | 0.0519 | NS |
| IL-6 - Saline vs Sepsis | |  |  |  |  |  |
|  | 1 | 4 | 3 |  | 0.3449 | NS |
|  |  |  |  |  |  |  |
| **Figure 4J.** Neocortex – P8 | |  |  |  |  |  |
| Mann Whitney test | P value | U | n | Unpaired t test | P value |  |
| TNF-alpha - Saline vs Sepsis | |  |  |  |  |  |
|  | 0.05 | 0 | 3 |  | 0.0001 | * |
| IL-1 beta - Saline vs Sepsis | |  |  |  |  |  |
|  | 1 | 4 | 3 |  | 0.4904 | NS |
| IL-6 - Saline vs Sepsis | |  |  |  |  |  |
|  | 0.05 | 0 | 3 |  | 0.0168 | * |
|  |  |  |  |  |  |  |
| **Figure 4K.** Frontal Cortex – P8 | |  |  |  |  |  |
| Mann Whitney test | P value | U | n | Unpaired t test | P value |  |
| TNF-alpha - Saline vs Sepsis | |  |  |  |  |  |
|  | 0.05 | 0 | 3 |  | 0.0149 | * |
| IL-1 beta - Saline vs Sepsis | |  |  |  |  |  |
|  | 0.05 | 0 | 3 |  | 0.0086 | * |
| IL-6 - Saline vs Sepsis | |  |  |  |  |  |
|  | 0.05 | 0 | 3 |  | 0.0055 | * |
|  |  |  |  |  |  |  |
| **Figure 4L.** Cerebellum – P8 | |  |  |  |  |  |
| Mann Whitney test | P value | U | n | Unpaired t test | P value |  |
| TNF-alpha - Saline vs Sepsis | |  |  |  |  |  |
|  | 0.2 | 2 | 3 |  | 0.1675 | NS |
| IL-1 beta - Saline vs Sepsis | |  |  |  |  |  |
|  | 0.05 | 0 | 3 |  | 0,0036 | * |
| IL-6 - Saline vs Sepsis | |  |  |  |  |  |
|  | 0.2533 | 2.5 | 3 |  | 0.3158 | NS |
|  |  |  |  |  |  |  |
| **Figure 5A.** Synaptophysin Levels - Hippocampus – P30 | | | |  |  |  |
| Mann Whitney test | P value | U | n | Unpaired t test | P value |  |
| Saline vs Sepsis | 0.3357 | 19 | 7 |  | 0.0873 | NS |
|  |  |  |  |  |  |  |
| **Figure 5B.** Synaptophysin Levels - Neocortex – P30 | | | |  |  |  |
| Mann Whitney test | P value | U | n | Unpaired t test | P value |  |
| Saline vs Sepsis | 0.0076 | 3 | 6 |  | 0.0022 | * |
|  |  |  |  |  |  |  |
| **Figure 5C.** Synaptophysin Levels - Frontal Cortex – P30 | | | |  |  |  |
| Mann Whitney test | P value | U | n | Unpaired t test | P value |  |
| Saline vs Sepsis | 0.1 | 1 | 3 |  | 0.1418 | NS |
|  |  |  |  |  |  |  |
| **Figure 5D.** Synaptophysin Levels - Cerebellum – P30 | | | |  |  |  |
| Mann Whitney test | P value | U | n | Unpaired t test | P value |  |
| Saline vs Sepsis | 0.6857 | 6 | 4 |  | 0.4097 | NS |
|  |  |  |  |  |  |  |
| **Figure 5E.** PSD95 Levels - Hippocampus – P30 | | |  |  |  |  |
| Mann Whitney test | P value | U | n | Unpaired t test | P value |  |
| Saline vs Sepsis | 0.05 | 0 | 3 |  | 0.0223 | * |
|  |  |  |  |  |  |  |
| **Figure 5F.** PSD95 Levels - Neocortex – P30 | | |  |  |  |  |
| Mann Whitney test | P value | U | n | Unpaired t test | P value |  |
| Saline vs Sepsis | 0.05 | 0 | 3 |  | 0.0241 | * |
|  |  |  |  |  |  |  |
| **Figure 5G.** PSD95 Levels - Frontal Cortex – P30 | | |  |  |  |  |
| Mann Whitney test | P value | U | n | Unpaired t test | P value |  |
| Saline vs Sepsis | 0.05 | 0 | 3 |  | 0.0291 | * |
|  |  |  |  |  |  |  |
| **Figure 5H.** PSD95 Levels - Cerebellum – P30 | | |  |  |  |  |
| Mann Whitney test | P value | U | n | Unpaired t test | P value |  |
| Saline vs Sepsis | 0.3429 | 6 | 4 |  | 0.3359 | NS |
|  |  |  |  |  |  |  |
| **Figure 5I.** Hippocampus – P30 | |  |  |  |  |  |
| Mann Whitney test | P value | U | n | Unpaired t test | P value |  |
| TNF-alpha - Saline vs Sepsis | |  |  |  |  |  |
|  | 0.05 | 0 | 3 |  | 0.0472 | * |
| IL-1 beta - Saline vs Sepsis | |  |  |  |  |  |
|  | 0.2 | 2 | 3 |  | 0.2113 | NS |
| IL-6 - Saline vs Sepsis | |  |  |  |  |  |
|  | 0.329 | 3 | 3 |  | 0.1588 | NS |
|  |  |  |  |  |  |  |
| **Figure 5J.** Neocortex – P30 | |  |  |  |  |  |
| Mann Whitney test | P value | U | n | Unpaired t test | P value |  |
| TNF-alpha - Saline vs Sepsis | |  |  |  |  |  |
|  | 0.05 | 0 | 3 |  | 0.0192 | * |
| IL-1 beta - Saline vs Sepsis | |  |  |  |  |  |
|  | 0.05 | 0 | 3 |  | 0.014 | * |
| IL-6 - Saline vs Sepsis | |  |  |  |  |  |
|  | 0.05 | 0 | 3 |  | 0.0003 | * |
|  |  |  |  |  |  |  |
| **Figure 5K.** Frontal Cortex – P30 | |  |  |  |  |  |
| Mann Whitney test | P value | U | n | Unpaired t test | P value |  |
| TNF-alpha - Saline vs Sepsis | |  |  |  |  |  |
|  | 0.05 | 0 | 3 |  | 0.0026 | * |
| IL-1 beta - Saline vs Sepsis | |  |  |  |  |  |
|  | 0.05 | 0 | 3 |  | 0.0137 | * |
| IL-6 - Saline vs Sepsis | |  |  |  |  |  |
|  | 0.05 | 0 | 3 |  | 0.0258 | * |
|  |  |  |  |  |  |  |
| **Figure 5L.** Cerebellum – P30 | |  |  |  |  |  |
| Mann Whitney test | P value | U | n | Unpaired t test | P value |  |
| TNF-alpha - Saline vs Sepsis | |  |  |  |  |  |
|  | 0.05 | 0 | 3 |  | 0.0315 | * |
| IL-1 beta - Saline vs Sepsis | |  |  |  |  |  |
|  | 0.2 | 2 | 3 |  | 0.3503 | NS |
| IL-6 - Saline vs Sepsis | |  |  |  |  |  |
|  | 0.2 | 2 | 3 |  | 0.0925 | NS |
|  |  |  |  |  |  |  |
| **Figure 6A.** Synaptophysin Levels - Hippocampus – P60 | | | |  |  |  |
| Mann Whitney test | P value | U | n | Unpaired t test | P value |  |
| Saline vs Sepsis | 0.0087 | 6 | 7 |  | 0.0052 | * |
|  |  |  |  |  |  |  |
| **Figure 6B.** Synaptophysin Levels - Neocortex – P60 | | | |  |  |  |
| Mann Whitney test | P value | U | n | Unpaired t test | P value |  |
| Saline vs Sepsis | 0.004 | 4 | 5 |  | 0.0016 | * |
|  |  |  |  |  |  |  |
| **Figure 6C.** Synaptophysin Levels - Frontal Cortex – P60 | | | |  |  |  |
| Mann Whitney test | P value | U | n | Unpaired t test | P value |  |
| Saline vs Sepsis | 0.2 | 2 | 3 |  | 0.1725 | NS |
|  |  |  |  |  |  |  |
| **Figure 6D.** Synaptophysin Levels - Cerebellum – P60 | | | |  |  |  |
| Mann Whitney test | P value | U | n | Unpaired t test | P value |  |
| Saline vs Sepsis | 0.1714 | 4 | 4 |  | 0.1955 | NS |
|  |  |  |  |  |  |  |
| **Figure 6E.** PSD95 Levels - Hippocampus – P60 | | |  |  |  |  |
| Mann Whitney test | P value | U | n | Unpaired t test | P value |  |
| Saline vs Sepsis | 0.0143 | 0 | 4 |  | 0.0082 | * |
|  |  |  |  |  |  |  |
| **Figure 6F.** PSD95 Levels - Neocortex – P60 | | |  |  |  |  |
| Mann Whitney test | P value | U | n | Unpaired t test | P value |  |
| Saline vs Sepsis | 0.05 | 0 | 3 |  | 0.028 | * |
|  |  |  |  |  |  |  |
| **Figure 6G.** PSD95 Levels - Frontal Cortex – P60 | | |  |  |  |  |
| Mann Whitney test | P value | U | n | Unpaired t test | P value |  |
| Saline vs Sepsis | 0.05 | 0 | 3 |  | 0.0359 | * |
|  |  |  |  |  |  |  |
| **Figure 6H.** PSD95 Levels - Cerebellum – P60 | | |  |  |  |  |
| Mann Whitney test | P value | U | n | Unpaired t test | P value |  |
| Saline vs Sepsis | 0.35 | 3 | 3 |  | 0.317 | NS |
|  |  |  |  |  |  |  |
| **Figure 6I.** Hippocampus – P60 | |  |  |  |  |  |
| Mann Whitney test | P value | U | n | Unpaired t test | P value |  |
| TNF-alpha - Saline vs Sepsis | |  |  |  |  |  |
|  | 0.05 | 0 | 3 |  | 0.028 | * |
| IL-1 beta - Saline vs Sepsis | |  |  |  |  |  |
|  | 0.05 | 0 | 3 |  | 0.0203 | * |
| IL-6 - Saline vs Sepsis | |  |  |  |  |  |
|  | 0.05 | 0 | 3 |  | 0.0056 | * |
|  |  |  |  |  |  |  |
| **Figure 6J.** Neocortex – P60 | |  |  |  |  |  |
| Mann Whitney test | P value | U | n | Unpaired t test | P value |  |
| TNF-alpha - Saline vs Sepsis | |  |  |  |  |  |
|  | 0.05 | 0 | 3 |  | 0.0074 | * |
| IL-1 beta - Saline vs Sepsis | |  |  |  |  |  |
|  | 0.05 | 0 | 3 |  | 0.0047 | * |
| IL-6 - Saline vs Sepsis | |  |  |  |  |  |
|  | 0.05 | 0 | 3 |  | 0.0139 | * |
|  |  |  |  |  |  |  |
| **Figure 6K.** Frontal Cortex – P60 | |  |  |  |  |  |
| Mann Whitney test | P value | U | n | Unpaired t test | P value |  |
| TNF-alpha - Saline vs Sepsis | |  |  |  |  |  |
|  | 0,2 | 2 | 3 |  | 0.096 | NS |
| IL-1 beta - Saline vs Sepsis | |  |  |  |  |  |
|  | 0.5 | 4 | 3 |  | 0.4888 | NS |
| IL-6 - Saline vs Sepsis | |  |  |  |  |  |
|  | 0.35 | 3 | 3 |  | 0.1742 | NS |
|  |  |  |  |  |  |  |
| **Figure 6L.** Cerebellum – P60 | |  |  |  |  |  |
| Mann Whitney test | P value | U | n | Unpaired t test | P value |  |
| TNF-alpha - Saline vs Sepsis | |  |  |  |  |  |
|  | 0.05 | 0 | 3 |  | 0.0423 | * |
| IL-1 beta - Saline vs Sepsis | |  |  |  |  |  |
|  | 0.35 | 3 | 3 |  | 0.2449 | NS |
| IL-6 - Saline vs Sepsis | |  |  |  |  |  |
|  | 0.2 | 2 | 3 |  | 0.1578 | NS |
|  |  |  |  |  |  |  |
| **Figure 7A**. Escape latency - Morris Water Maze – P30 | | | |  |  |  |
| Source of Variation | % of total variation | P value | t=1.947 |  |  |  |
| Interaction | 3.31 | 0.0632 | df=6 |  |  |  |
| Row Factor | 44.58 | 0.0182 |  |  |  |  |
| Time | 30.25 | P<0.0001 |  |  |  |  |
| Subjects (matching) | 194.453 | 0.0040 |  |  |  |  |
|  |  |  |  |  |  |  |
| Source of Variation | P value summary | Significant? |  |  |  |  |
| Interaction | ns | No |  |  |  |  |
| Row Factor | * | Yes |  |  |  |  |
| Time | *** | Yes |  |  |  |  |
| Subjects (matching) | ** | Yes |  |  |  |  |
|  |  |  |  |  |  |  |
| **Figure 7B.** Escape latency - Morris Water Maze – P60 | | | |  |  |  |
| Source of Variation | % of total variation | P value | t=1.064 |  |  |  |
| Interaction | 4.87 | 0.0993 | df=6 |  |  |  |
| Time | 53.69 | P<0.0001 |  |  |  |  |
| Column Factor | 11.04 | 0.2375 |  |  |  |  |
| Subjects (matching) | 229.371 | 0.0012 |  |  |  |  |
|  |  |  |  |  |  |  |
| Source of Variation | P value summary | Significant? |  |  |  |  |
| Interaction | ns | No |  |  |  |  |
| Time | *** | Yes |  |  |  |  |
| Column Factor | ns | No |  |  |  |  |
| Subjects (matching) | ** | Yes |  |  |  |  |
|  |  |  |  |  |  |  |
| **Figure 7C.** Time spent on hidden quadrant - Moris Water Maze – P30 | | | | |  |  |
| Mann Whitney test | P value | U | n | Unpaired t test | P value |  |
| Saline vs Sepsis | 0.2973 | 28 | 9 |  | 0.1487 | NS |
|  |  |  |  |  |  |  |
| **Figure 7D.** Time spent on hidden quadrant - Moris Water Maze – P60 | | | | |  |  |
| Mann Whitney test | P value | U | n | Unpaired t test | P value |  |
| Saline vs Sepsis | 0.0022 | 9 | 9 |  | 0.002 | * |
|  |  |  |  |  |  |  |
| **Figure 7E.** Fear conditioning memory test – P30 | | |  |  |  |  |
| Mann Whitney test | P value | U | n | Unpaired t test | P value |  |
| Saline vs Sepsis | 0.0001 | 3 | 10 |  | 0.0001 | * |
|  |  |  |  |  |  |  |
| **Figure 7F.** Fear conditioning memory test – P30 | | |  |  |  |  |
| Mann Whitney test | P value | U | n | Unpaired t test | P value |  |
| Saline vs Sepsis | 0.0005 | 3.5 | 9 |  | 0.0001 | * |
|  |  |  |  |  |  |  |
| **Figure 8A.** Forced swim – P30 | |  |  |  |  |  |
| Mann Whitney test | P value | U | n | Unpaired t test | P value |  |
| Saline vs Sepsis | 0.0086 | 16 | 9 |  | 0.0058 | * |
|  |  |  |  |  |  |  |
| **Figure 8B.** Tail Suspension – P30 | | |  |  |  |  |
| Mann Whitney test | P value | U | n | Unpaired t test | P value |  |
| Saline vs Sepsis | 0.0014 | 3 | 9 |  | 0.0005 | * |
|  |  |  |  |  |  |  |
| **Figure 8C.** Sucrose preference – P30 | | |  |  |  |  |
| Mann Whitney test | P value | U | n | Unpaired t test | P value |  |
| Saline vs Sepsis | 0.3788 | 28 | 9 |  | 0.486 | NS |
|  |  |  |  |  |  |  |
| **Figure 8D.** Forced swim – P60 | |  |  |  |  |  |
| Mann Whitney test | P value | U | n | Unpaired t test | P value |  |
| Saline vs Sepsis | 0.0002 | 6 | 11 |  | 0.0001 | * |
|  |  |  |  |  |  |  |
| **Figure 8E.** Tail Suspension – P60 | |  |  |  |  |  |
| Mann Whitney test | P value | U | n | Unpaired t test | P value |  |
| Saline vs Sepsis | 0.007 | 7 | 9 |  | 0.0036 | * |
|  |  |  |  |  |  |  |
| **Figure 8F.** Sucrose preference – P60 | | |  |  |  |  |
| Mann Whitney test | P value | U | n | Unpaired t test | P value |  |
| Saline vs Sepsis | 0.0823 | 5 | 9 |  | 0.0561 | NS |
|  |  |  |  |  |  |  |
| **Additional file 3A.** Liver – P2 | |  |  |  |  |  |
| Mann Whitney test | P value | U | n | Unpaired t test | P value |  |
| TNF-alpha - Saline vs Sepsis | |  |  |  |  |  |
|  | 0.05 | 0 | 3 |  | 0.0148 | * |
| IL-1 beta - Saline vs Sepsis | |  |  |  |  |  |
|  | 0.05 | 0 | 3 |  | 0.0096 | * |
| IL-6 - Saline vs Sepsis | |  |  |  |  |  |
|  | 0.05 | 0 | 3 |  | 0.0281 | * |
|  |  |  |  |  |  |  |
| **Additional file 3B.** Lung – P2 | |  |  |  |  |  |
| Mann Whitney test | P value | U | n | Unpaired t test | P value |  |
| TNF-alpha - Saline vs Sepsis | |  |  |  |  |  |
|  | 0.0286 | 1 | 3 |  | 0.0084 | * |
| IL-1 beta - Saline vs Sepsis | |  |  |  |  |  |
|  | 0.1486 | 11 | 3 |  | 0.0617 | NS |
| IL-6 - Saline vs Sepsis | |  |  |  |  |  |
|  | 0.0185 | 4.5 | 3 |  | 0.0136 | * |
|  |  |  |  |  |  |  |
| **Additional file 3C.** Brain – P2 | |  |  |  |  |  |
| Mann Whitney test | P value | U | n | Unpaired t test | P value |  |
| TNF-alpha - Saline vs Sepsis | |  |  |  |  |  |
|  | 0.05 | 0 | 3 |  | 0.013 | * |
| IL-1 beta - Saline vs Sepsis | |  |  |  |  |  |
|  | 0.05 | 0 | 3 |  | 0.012 | * |
| IL-6 - Saline vs Sepsis | |  |  |  |  |  |
|  | 5 | 4 | 3 |  | 0.3157 | NS |
|  |  |  |  |  |  |  |
| **Additional file 3D** Liver – P8 | |  |  |  |  |  |
| Mann Whitney test | P value | U | n | Unpaired t test | P value |  |
| TNF-alpha - Saline vs Sepsis | |  |  |  |  |  |
|  | 0.4 | 2 | 3 |  | 0.1699 | NS |
| IL-1 beta - Saline vs Sepsis | |  |  |  |  |  |
|  | 0.1 | 1 | 3 |  | 0.0657 | NS |
| IL-6 - Saline vs Sepsis | |  |  |  |  |  |
|  | 0.2 | 2 | 3 |  | 0.1332 | NS |
|  |  |  |  |  |  |  |
| **Additional file 3E.** Lung – P8 | |  |  |  |  |  |
| Mann Whitney test | P value | U | n | Unpaired t test | P value |  |
| TNF-alpha - Saline vs Sepsis | |  |  |  |  |  |
|  | 0.05 | 0 | 3 |  | 0.0037 | * |
| IL-1 beta - Saline vs Sepsis | |  |  |  |  |  |
|  | 0.4 | 2 | 3 |  | 0.1056 | NS |
| IL-6 - Saline vs Sepsis | |  |  |  |  |  |
|  | 0.329 | 3 | 3 |  | 0.1588 | NS |
|  |  |  |  |  |  |  |
| **Additional file 3F.** Brain – P8 | |  |  |  |  |  |
| Mann Whitney test | P value | U | n | Unpaired t test | P value |  |
| TNF-alpha - Saline vs Sepsis | |  |  |  |  |  |
|  | 0.05 | 0 | 3 |  | 0.0111 | * |
| IL-1 beta - Saline vs Sepsis | |  |  |  |  |  |
|  | 0.35 | 3 | 3 |  | 0.2209 | NS |
| IL-6 - Saline vs Sepsis | |  |  |  |  |  |
|  | 0.05 | 0 | 3 |  | 0.0218 | * |
|  |  |  |  |  |  |  |
| **Additional file 4A.** Liver – P30 | |  |  |  |  |  |
| Mann Whitney test | P value | U | n | Unpaired t test | P value |  |
| TNF-alpha - Saline vs Sepsis | |  |  |  |  |  |
|  | 0.8571 | 5 | 3 |  | 0.4018 | NS |
| IL-1 beta - Saline vs Sepsis | |  |  |  |  |  |
|  | 0.2 | 3 | 3 |  | 0.1714 | NS |
| IL-6 - Saline vs Sepsis | |  |  |  |  |  |
|  | 0.3143 | 4 | 3 |  | 0.1957 | NS |
|  |  |  |  |  |  |  |
| **Additional file 4B.** Lung – P30 | |  |  |  |  |  |
| Mann Whitney test | P value | U | n | Unpaired t test | P value |  |
| TNF-alpha - Saline vs Sepsis | |  |  |  |  |  |
|  | 0.35 | 3 | 3 |  | 0.3564 | NS |
| IL-1 beta - Saline vs Sepsis | |  |  |  |  |  |
|  | 0.8571 | 5 | 3 |  | 0.4373 | NS |
| IL-6 - Saline vs Sepsis | |  |  |  |  |  |
|  | 0.3143 | 3 | 3 |  | 0.2441 | NS |
|  |  |  |  |  |  |  |
| **Additional file 4C.** Brain – P30 | |  |  |  |  |  |
| Mann Whitney test | P value | U | n | Unpaired t test | P value |  |
| TNF-alpha - Saline vs Sepsis | |  |  |  |  |  |
|  | 0.05 | 0 | 3 |  | 0.018 | * |
| IL-1 beta - Saline vs Sepsis | |  |  |  |  |  |
|  | 0.05 | 0 | 3 |  | 0.0112 | * |
| IL-6 - Saline vs Sepsis | |  |  |  |  |  |
|  | 0.05 | 0 | 3 |  | 0.0001 | * |
|  |  |  |  |  |  |  |
| **Additional file 4D** Liver – P60 | |  |  |  |  |  |
| Mann Whitney test | P value | U | n | Unpaired t test | P value |  |
| TNF-alpha - Saline vs Sepsis | |  |  |  |  |  |
|  | 0.4286 | 5 | 3 |  | 0.334 | NS |
| IL-1 beta - Saline vs Sepsis | |  |  |  |  |  |
|  | 0.35 | 3 | 3 |  | 0.2218 | NS |
| IL-6 - Saline vs Sepsis | |  |  |  |  |  |
|  | 0.2 | 2 | 3 |  | 0.0871 | NS |
|  |  |  |  |  |  |  |
| **Additional file 4E.** Lung – P60 | |  |  |  |  |  |
| Mann Whitney test | P value | U | n | Unpaired t test | P value |  |
| TNF-alpha - Saline vs Sepsis | |  |  |  |  |  |
|  | 0.5 | 4 | 3 |  | 0.4878 | NS |
| IL-1 beta - Saline vs Sepsis | |  |  |  |  |  |
|  | 0.2 | 2 | 3 |  | 0.1456 | NS |
| IL-6 - Saline vs Sepsis | |  |  |  |  |  |
|  | 1 | 4 | 3 |  | 0.451 | NS |
|  |  |  |  |  |  |  |
| **Additional file 4F.** Brain – P60 | |  |  |  |  |  |
| Mann Whitney test | P value | U | n | Unpaired t test | P value |  |
| TNF-alpha - Saline vs Sepsis | |  |  |  |  |  |
|  | 0.05 | 0 | 3 |  | 0.0435 | * |
| IL-1 beta - Saline vs Sepsis | |  |  |  |  |  |
|  | 0.05 | 0 | 3 |  | 0.0001 | * |
| IL-6 - Saline vs Sepsis | |  |  |  |  |  |
|  | 0.05 | 0 | 3 |  | 0.0094 | * |
|  |  |  |  |  |  |  |
| **Additional file 5A.** Time spent - Elevated Pluz Maze – P30 | | | |  |  |  |
| Mann Whitney test | P value | U | n | Unpaired t test | P value |  |
| Saline vs Sepsis | 0.4261 | 31 | 9 |  | 0.213 | NS |
|  |  |  |  |  |  |  |
| **Additional file 5B.** Entires - Elevated Pluz Maze – P30 | | | |  |  |  |
| Mann Whitney test | P value | U | n | Unpaired t test | P value |  |
| Saline vs Sepsis | 0.0454 | 21.5 | 9 |  | 0.0557 | * |
|  |  |  |  |  |  |  |
| **Additional file 5C.** Time spent - Elevated Pluz Maze – P60 | | | |  |  |  |
| Mann Whitney test | P value | U | n | Unpaired t test | P value |  |
| Saline vs Sepsis | 0.4894 | 32 | 9 |  | 0.2447 | NS |
|  |  |  |  |  |  |  |
| **Additional file 5D.** Entires - Elevated Pluz Maze – P60 | | | |  |  |  |
| Mann Whitney test | P value | U | n | Unpaired t test | P value |  |
| Saline vs Sepsis | 0.3433 | 35.5 | 9 |  | 0.4284 | NS |
|  |  |  |  |  |  |  |
| **Additional file 5E.** Time spent on light box - Light/Dark Box – P30 | | | |  |  |  |
| Mann Whitney test | P value | U | n | Unpaired t test | P value |  |
| Saline vs Sepsis | 0.2224 | 26 | 9 |  | 0.1112 | NS |
|  |  |  |  |  |  |  |
| **Additional file 5F.** Entrances on light box - Light/Dark Box – P30 | | | |  |  |  |
| Mann Whitney test | P value | U | n | Unpaired t test | P value |  |
| Saline vs Sepsis | 0.3961 | 3.5 | 9 |  | 0.1981 | NS |
|  |  |  |  |  |  |  |
| **Additional file 5G.** Light/Dark transition - Light/Dark Box – P30 | | | |  |  |  |
| Mann Whitney test | P value | U | n | Unpaired t test | P value |  |
| Saline vs Sepsis | 0.2224 | 26 | 9 |  | 0.1112 | NS |
|  |  |  |  |  |  |  |
| **Additional file 5H.** Time spent on light box - Light/Dark Box – P60 | | | |  |  |  |
| Mann Whitney test | P value | U | n | Unpaired t test | P value |  |
| Saline vs Sepsis | 0.6485 | 43 | 9 |  | 0.3243 | NS |
|  |  |  |  |  |  |  |
| **Additional file 5I.** Entrances on light box - Light/Dark Box – P60 | | | |  |  |  |
| Mann Whitney test | P value | U | n | Unpaired t test | P value |  |
| Saline vs Sepsis | 0.6486 | 38 | 9 |  | 0.3243 | NS |
|  |  |  |  |  |  |  |
| **Additional file 5J.** Light/Dark transition - Light/Dark Box – P60 | | | |  |  |  |
| Mann Whitney test | P value | U | n | Unpaired t test | P value |  |
| Saline vs Sepsis | 0.8688 | 41.5 | 9 |  | 0.4344 | NS |
| * P < 0.05 | ** P<0.01 | *** P<0.001 |  | NS (non significant) |  |  |
