# Supplementary material for: Inflammatory, synaptic, motor, and behavioral alterations induced by gestational sepsis on the offspring at different stages of life
Source: J Neuroinflammation. 2021 Feb 25;18:60. doi: 10.1186/s12974-021-02106-1 (PMC7905683; doi:10.1186/s12974-021-02106-1)
Supplement: Supplementary file 2 — Additional file 2. Additional table 2 Clinical conditions of Gestational Sepsis. [file 12974_2021_2106_MOESM2_ESM.docx]

| **Additional table 2 - Clinical conditions of Gestational Sepsis** | | | | |  |
| --- | --- | --- | --- | --- | --- |
| 1. Number of offspring per litter | |  |  |  |  |
| Saline | Mean ± SEM | 11.56 ± 0.7837 N=37 | | P= 0.0029 |  |
| Sepsis | Mean ± SEM | 8.333 ± 0.6455 N=56 | | t=3.174 |  |
|  |  |  |  | df=16 |  |
| 2. Percentage of miscarriage | |  |  |  |  |
| Groups |  |  |  |  |  |
| Saline | Number of miscarriage= 1 | | Total number of animals= 37 | | |
| Sepsis | Number of miscarriage = 17 | | Total number of animals= 56 | | |
|  |  |  |  |  |  |
| Saline | % of miscarriage = 2,7% | |  |  |  |
| Sepsis | % of miscarriage = 30% | |  |  |  |
|  |  |  |  |  |  |
| 3. Offspring weight of P30 | |  |  |  |  |
| Saline | Mean ± SEM | 23.71 ± 0.4734 |  |  |  |
| Sepsis | Mean ± SEM | 23.67 ± 0.6629 |  |  |  |
| Mann Whitney test | P value | U | n | Unpaired t test | P value |
| Saline vs Sepsis | 0.4685 | 159 | 20 |  | 0.4823 |
|  |  |  |  |  |  |
| 4. Offspring weight of P60 | |  |  |  |  |
| Saline | Mean ± SEM | 42.71 ± 0.7911 |  |  |  |
| Sepsis | Mean ± SEM | 43.06 ± 0.6655 |  |  |  |
| Mann Whitney test | P value | U | n | Unpaired t test | P value |
| Saline vs Sepsis | 0.1017 | 152.5 | 20 |  | 0.1888 |
