# Supplementary material for: Inflammatory, synaptic, motor, and behavioral alterations induced by gestational sepsis on the offspring at different stages of life
Source: J Neuroinflammation. 2021 Feb 25;18:60. doi: 10.1186/s12974-021-02106-1 (PMC7905683; doi:10.1186/s12974-021-02106-1)
Supplement: Supplementary file 3 — Additional file 3. Pre-natal exposure to sepsis induces neonatal systemic inflammatory response. (A-C) TNF-α, IL-1β and IL-6 levels analyzed by ELISA in the liver (A), lungs (B) and brain (C) P2. Each bar is the mean +/- SEM from at least 5 animals. (D-F) TNF-α, IL-1β and IL-6 levels analyzed by ELISA in the liver (D), lungs (E) and brain (F) P8. Each bar is the mean +/- SEM from at least 5 animals. * p<0.05 comparing saline to sepsis. [file 12974_2021_2106_MOESM3_ESM.docx]

**
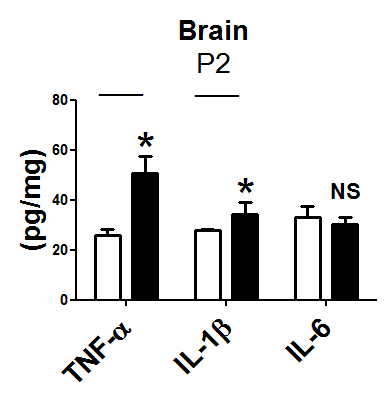

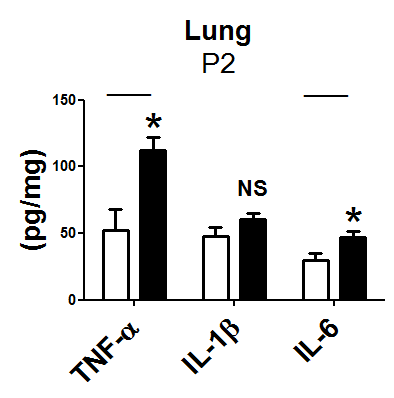

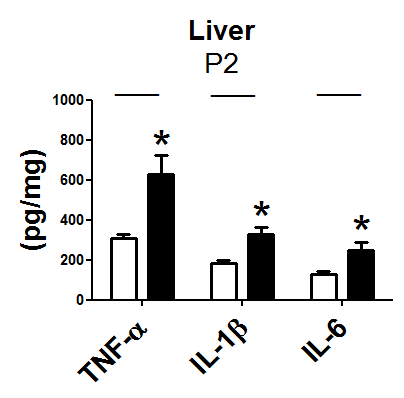
 A B C**

**
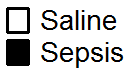
**

**
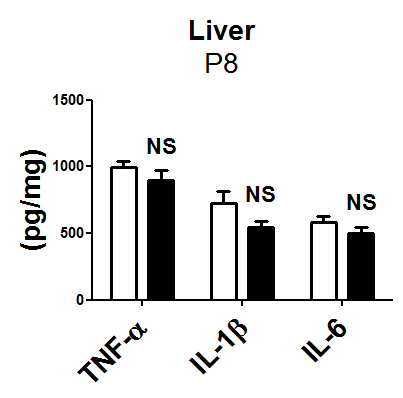
 D
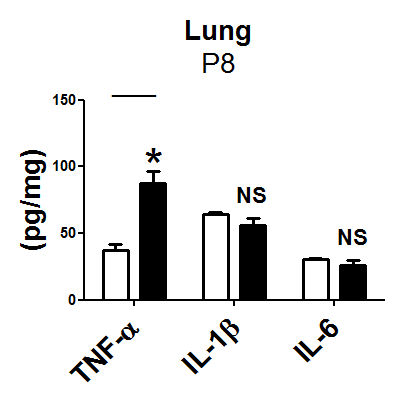
E
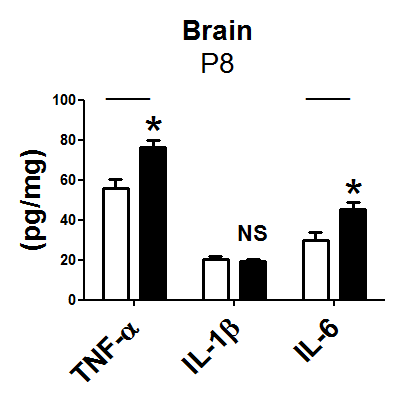
 F**

**Additional File 3**: Pre-natal exposure to sepsis induces neonatal systemic inflammatory response. **(A-C)** TNF-α, IL-1β and IL-6 levels analyzed by ELISA in the liver (A), lungs (B) and brain (C) P2. Each bar is the mean +/- SEM from at least 5 animals. **(D-F)** TNF-α, IL-1β and IL-6 levels analyzed by ELISA in the liver (D), lungs (E) and brain (F) P8. Each bar is the mean +/- SEM from at least 5 animals. * p<0.05 comparing saline to sepsis.
