# Supplementary material for: Inflammatory, synaptic, motor, and behavioral alterations induced by gestational sepsis on the offspring at different stages of life
Source: J Neuroinflammation. 2021 Feb 25;18:60. doi: 10.1186/s12974-021-02106-1 (PMC7905683; doi:10.1186/s12974-021-02106-1)
Supplement: Supplementary file 4 — Additional file 4. Pre-natal exposure to sepsis impacts in systemic inflammatory response in young and adult offspring. (A-C) TNF-α, IL-1β and IL-6 levels analyzed by ELISA in the liver (A), lungs (B) and brain (C) P30. Each bar is the mean +/- SEM from at least 5 animals. (D-F) TNF-α, IL-1β and IL-6 levels analyzed by ELISA in the liver (D), lungs (E) and brain (F) P60. Each bar is the mean +/- SEM from at least 5 animals. * p<0.05 comparing saline to sepsis. [file 12974_2021_2106_MOESM4_ESM.docx]

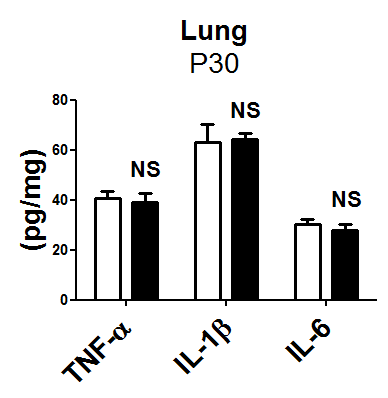

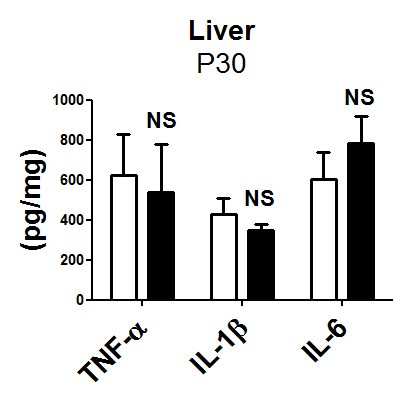
 A B
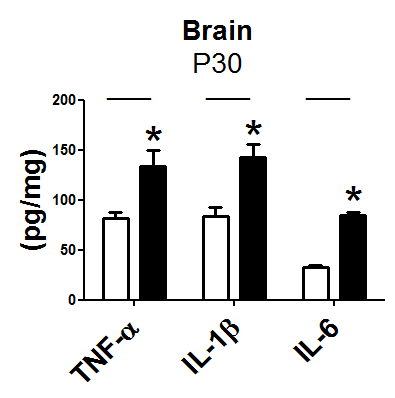
C


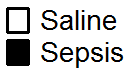


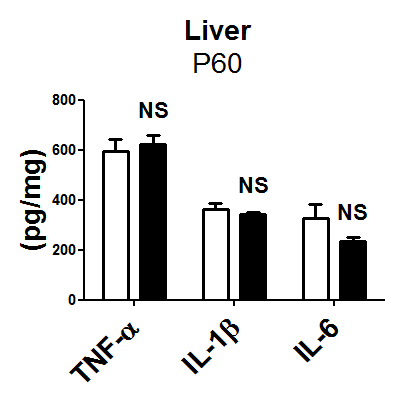
 D
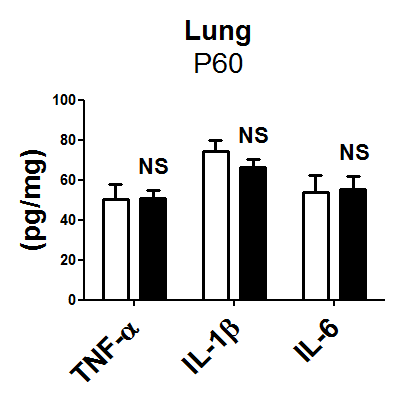
 E
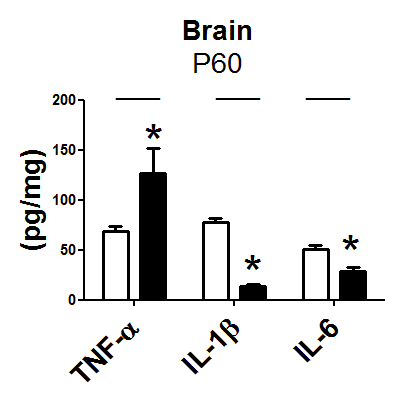
 F

**Additional File 4**: Pre-natal exposure to sepsis impacts in systemic inflammatory response in young and adult offspring. **(A-C)** TNF-α, IL-1β and IL-6 levels analyzed by ELISA in the liver (A), lungs (B) and brain (C) P30. Each bar is the mean +/- SEM from at least 5 animals. **(D-F)** TNF-α, IL-1β and IL-6 levels analyzed by ELISA in the liver (D), lungs (E) and brain (F) P60. Each bar is the mean +/- SEM from at least 5 animals. * p<0.05 comparing saline to sepsis.
