# Supplementary material for: Inflammatory, synaptic, motor, and behavioral alterations induced by gestational sepsis on the offspring at different stages of life
Source: J Neuroinflammation. 2021 Feb 25;18:60. doi: 10.1186/s12974-021-02106-1 (PMC7905683; doi:10.1186/s12974-021-02106-1)
Supplement: Supplementary file 5 — Additional file 5. Effects of gestational sepsis in young and adult offspring anxiety-like behavior of P30 and P60. (A-B) The elevated plus maze data were measured and compared of P30 offspring by the time spent in open arms (A) and total number of entries into open arms (B). Each point is the mean +/- SEM n= 9-12. (C-D) Elevated plus maze of P60 offspring analyzed by the time spent in open arms (C) and total number of entries into open arms (D). Each point is the mean +/- SEM n= 9-12. (E-J) The light/dark box test exposed the P30 offspring (E-J) and P60 (H-J) offspring to an aversive ambient (lit compartment) and evaluate the time spent on lit compartment (E, H), number of entrances on the lit compartment (F, I), and time spent in transitions between the lit and dark compartments (G, J). Each bar is the mean +/- SEM from n= 9-12 * p<0.05 comparing saline to sepsis. [file 12974_2021_2106_MOESM5_ESM.docx]

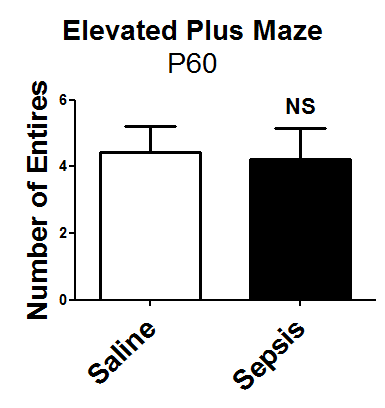
 A
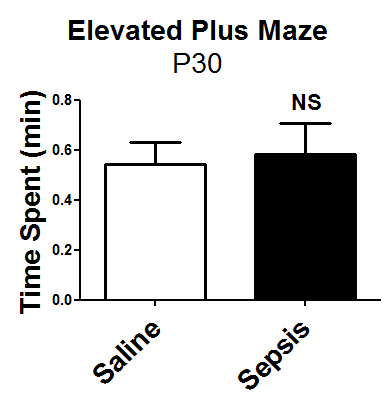
 B
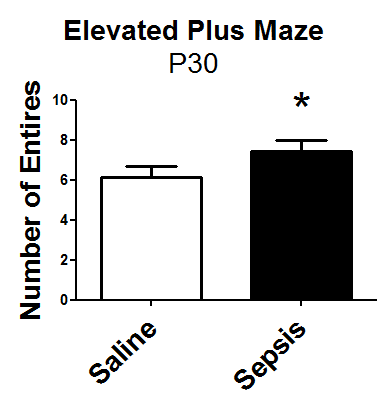
 C
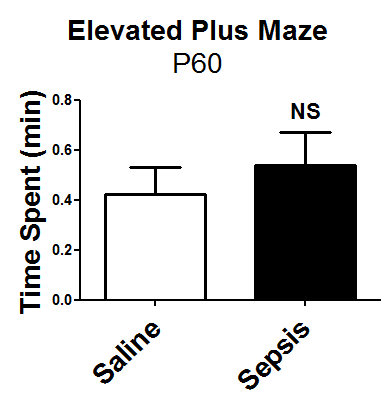
 D


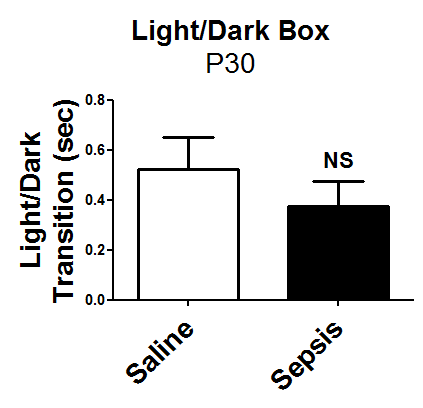

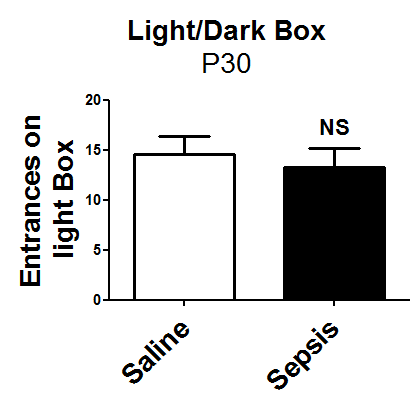

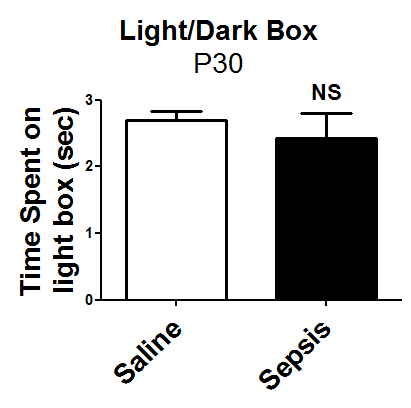
 E F G


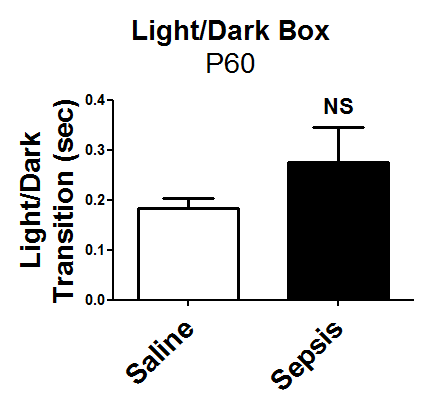

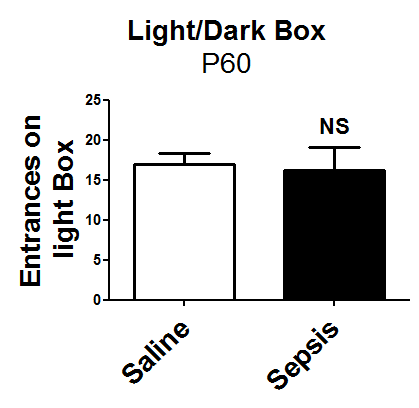

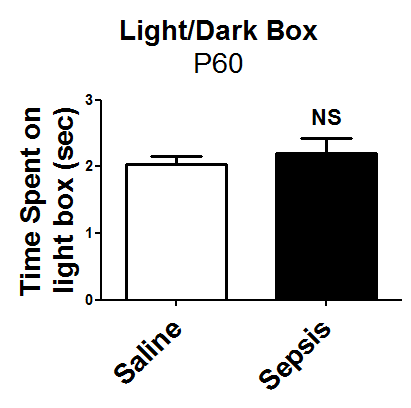
 H I J

**Additional File 5**: Effects of gestational sepsis in young and adult offspring anxiety-like behavior of P30 and P60. **(A-B)** The elevated plus maze data were measured and compared of P30 offspring by the time spent in open arms (A) and total number of entries into open arms (B). Each point is the mean +/- SEM n= 9-12. **(C-D)** Elevated plus maze of P60 offspring analyzed by the time spent in open arms (C) and total number of entries into open arms (D). Each point is the mean +/- SEM n= 9-12. **(E-J)** The light/dark box test exposed the P30 offspring (E-J) and P60 (H-J) offspring to an aversive ambient (lit compartment) and evaluate the time spent on lit compartment (E, H), number of entrances on the lit compartment (F, I), and time spent in transitions between the lit and dark compartments (G, J). Each bar is the mean +/- SEM from n= 9-12 * p<0.05 comparing saline to sepsis.
